# Pancreatogenic Diabetes: Triggering Effects of Alcohol and HIV

**DOI:** 10.3390/biology10020108

**Published:** 2021-02-03

**Authors:** Moses New-Aaron, Murali Ganesan, Raghubendra Singh Dagur, Kusum K. Kharbanda, Larisa Y. Poluektova, Natalia A. Osna

**Affiliations:** 1 Department of Environmental Health, Occupational Health and Toxicology, University of Nebraska Medical Center, Omaha, NE 68198, USA; 2 Veteran Affairs Nebraska—Western Iowa Health Care System, Omaha, NE 68105, USA; murali.ganesan@unmc.edu (M.G.); raghu.dagur@unmc.edu (R.S.D.); kkharbanda@unmc.edu (K.K.K.); 3 Department of Internal Medicine, University of Nebraska Medical Center, Omaha, NE 68198, USA; 4 Department of Pharmacology and Experimental Neuroscience, University of Nebraska Medical Center, Omaha, NE 68198, USA; lpoluekt@unmc.edu

**Keywords:** HIV, pancreatic acinar cells, pancreatic stellate cells, ethanol metabolites, pancreatitis, diabetes mellitus, pancreatotoxicity

## Abstract

**Simple Summary:**

Did you know that HIV may directly cause organ damage despite the effects of highly active antiretroviral therapy (HAART)? Due to the potency of current HAART, this may look questionable; however, excessive alcohol use may increase the risk of HIV-induced organ damage. While the most implicated organ in the gastrointestinal system is the liver, the pancreas may also be affected. In this study, we aimed to disclose the mechanisms of pancreatitis in alcohol-abusing HIV patients, which is crucial for developing an effective therapeutic strategy. From the literature, we found that alcohol-induced intracellular zymogen activation was mediated by calcium and lysosome hydrolases leading to acinar necrosis. Similarly, HIV entry into pancreatic acinar cells mediates ER and oxidative stress, which triggers acinar necrosis. Infiltration of immune cells has also been reported to induce necrosis. Therefore, effective therapeutic regimens for HIV and alcohol-induced pancreatitis should inhibit HIV entry and ameliorate alcohol’s toxic effects on the pancreas.

**Abstract:**

Multiorgan failure may not be completely resolved among people living with HIV despite HAART use. Although the chances of organ dysfunction may be relatively low, alcohol may potentiate HIV-induced toxic effects in the organs of alcohol-abusing, HIV-infected individuals. The pancreas is one of the most implicated organs, which is manifested as diabetes mellitus or pancreatic cancer. Both alcohol and HIV may trigger pancreatitis, but the combined effects have not been explored. The aim of this review is to explore the literature for understanding the mechanisms of HIV and alcohol-induced pancreatotoxicity. We found that while premature alcohol-inducing zymogen activation is a known trigger of alcoholic pancreatitis, HIV entry through C-C chemokine receptor type 5 (CCR5) into pancreatic acinar cells may also contribute to pancreatitis in people living with HIV (PLWH). HIV proteins induce oxidative and ER stresses, causing necrosis. Furthermore, infiltrative immune cells induce necrosis on HIV-containing acinar cells. When necrotic products interact with pancreatic stellate cells, they become activated, leading to the release of both inflammatory and profibrotic cytokines and resulting in pancreatitis. Effective therapeutic strategies should block CCR5 and ameliorate alcohol’s effects on acinar cells.

## 1. Introduction

HIV remains a serious public health issue even 40 years after the first diagnosed AIDS case in the US. Approximately 76 million people have been infected with HIV since 1981 [1]. In 2018, 38 million people were infected globally [2]; 3% of these infections were in the United States, with an estimated 36,400 new infections [3]. Approximately 80,000 AIDS-related deaths were reported in 1992 [4], with a consistent decline to 5698 deaths in 2017 [5]. The decline in AIDS-related mortality is strongly associated with the emergence of highly active antiretroviral therapy [6,7,8,9], which has led to a significant increase in life expectancy [10,11,12]. However, as life expectancy of people living with HIV (PLWH) matches that of the general population [13], non-AIDS-related morbidities [12,14], such as cardiovascular disease [15], liver disease, suicide [16], diabetes [17,18,19,20,21,22,23] and alcohol abuse [24] begin to emerge. Diabetes is one of the leading causes of comorbidity among PLWH. This may also be a risk factor for the incidence of other cardiometabolic diseases [9,25]. The etiologies of diabetes in PLWH are multifactorial [26,27,28,29]. However, pancreatitis is one of the established risk factors for diabetes [30,31,32,33]. The incidence of pancreatitis is 35–800 times higher among AIDS patients—as compared to the general population [34,35,36]—and the incidence of diabetes from preexisting cases of pancreatitis is 10–83% [37]. Hence, the prevalence of diabetes among PLWH is 4.5–14%, which is higher than the 2% observed in the general population [38,39,40,41]. Although recent studies have implicated antiretroviral therapy as the major cause of pancreatitis in the context of HIV [42,43,44,45], this may be disputed [46,47,48] due to significant clinical evidence from the pre-HAART era, which shows that HIV itself may be a potent causative agent for pancreatitis [34,49,50]. Conflicting evidence on the pathogenesis of pancreatitis among PLWH [49] means that it is imperative to evaluate available scholarly evidence on the mechanisms that lead to it. Previous studies have reported strong correlations between alcohol abuse and other non-AIDS-related morbidities [50]. This suggests that alcohol plays a significant role in the pathogenesis of non-AIDS-related comorbidities. Alcohol use disorder (AUD) among PLWH is 2–3 times higher than the general population [50] and approximately 12% of PLWH are heavy drinkers [51]. While alcohol is one of the inducers of pancreatitis, pancreatitis usually requires other coexisting risk factors (e.g., HIV infection) to progress to diabetes [52]. The mechanisms of the toxic synergism between alcohol and HIV in pancreatic cells (acinar cells) that leads to pancreatitis have not been properly elucidated. Exploring the mechanisms of HIV- and alcohol-induced pancreatitis is fundamental for developing therapeutic regimens among alcohol-abusing HIV patients. Hence, this narrative review will uncover the role of alcohol in exacerbating necrosis in HIV-containing acinar cells, which becomes the basis for pancreatic damage.

Our objective is to elucidate the pivotal events leading to alcohol- and HIV-induced pancreatitis. We hypothesize that HIV entry and infectivity of acinar cells is potentiated by alcohol metabolites, which leads to the generation of oxidative stress and endoplasmic reticulum (ER) stress. This, in turn, results in necrosis, thereby triggering the activation of pancreatic stellate cells and progression to pancreatic damage. In this review, we will discuss the mechanisms leading to the toxic synergism between alcohol and HIV, which leads to pancreatic inflammation and damage.

## 2. Epidemiology of Pancreatitis and Diabetes

Pancreatitis is a localized inflammation of the pancreas commonly mediated by the premature activation of digestive enzymes retained in the pancreas. Even though this condition may resolve by itself within days, the persistence results in pancreatic dysfunction and failure of other remote organs/systems [49]. Pancreatitis occurs in two forms: acute and chronic. It was recently discovered that chronic pancreatitis is a consequence of repeated episodes of an acute case, indicating that both are the same disease at different stages [53]. A meta-analysis conducted by Xiao et al., reported the global, pooled incidences of pancreatitis as follows: acute pancreatitis, 34 cases/100,000; chronic pancreatitis, 10 cases/100,000; pancreatogenic diabetes mellitus, 6 cases/100,000 [54]. While the aforementioned rates reflect the combined incidence of pancreatitis, varying rates have been reported in different settings. For example, Albania (5.6/100,000) [55], Czech Republic (17/100000) [56], Germany (13/100000) [57] and the Netherlands (19.2/100,000) [58] reported lower incidences of acute pancreatitis, while Croatia (30.2/100,000) [59], Denmark (35/100,000) [60], Scotland (41.9/100,000) [61], Spain (67/100,000) [62], Finland (73.4/100,000) [63] and Poland (100/10,000) [64] have reported higher rates. Meanwhile, the global prevalence of pancreatitis has continued to increase. In 1990, the prevalent cases numbered approximately three million—and this rose to more than six million cases in 2017 [65]. While lifestyle factors have been implicated in the upsurge in the rates of pancreatitis [66], adequate case reports and access to quality data may be partly responsible for this notable rise. It suffices to say that the global burden of pancreatitis is a lingering GI problem.

With respect to rates in the US, data from the Nationwide Inpatient Sample (NIS) (which is the most robust database for all-payer in-patients and constitutes 85% of all hospital discharges) was queried for the prevalence of pancreatitis between 1988 and 2004. It revealed that while the average prevalence of acute pancreatitis was 49.2 cases/100,000, it was only 8.1/100,000 for chronic pancreatitis [67]. Peery et al., expanded the study on the burden of pancreatitis to include other high-quality national databases. They found that acute pancreatitis accounted for the majority of hospitalizations, at approximately 280,000 patients [68]. These elevated rates, both globally and in the US, explain why research on pancreatitis is of paramount importance.

The frequency of recurrent acute pancreatitis and consequent chronic pancreatitis was estimated recently in a systematic review of cohort studies with a minimum of one-year follow up. Interventional studies were not included in the study because interventions will alter the natural shift that occurs between acute and chronic pancreatitis. In that study, 21% of patients had recurrent acute pancreatitis and 36% developed chronic pancreatitis after initial acute pancreatitis [49]. The incidence of acute pancreatitis has been shown to lead to multiple organ/system dysfunctions, affecting endocrine, exocrine and even bone metabolism long after clinical resolution of pancreatitis [49].

Pancreatitis in any form has been frequently associated with diabetes. Data suggest that even patients with mild acute pancreatitis (i.e., most patients with acute pancreatitis) have at least a two-fold higher long-term risk of diabetes mellitus than people without a history of pancreatitis [30,31]. Hence, pancreatogenic diabetes mellitus is the aggravation of insulin deficiency induced by continuing inflammation and fibrosis of the exocrine tissues. This implies that chronic pancreatitis is an established precursor of diabetes [69]. A single-center cohort study conducted in China, in which 445 participants were diagnosed with chronic pancreatitis, revealed the frequency of diabetes development as 3.6%, starting from the onset of chronic pancreatitis. Furthermore, after one year of chronic pancreatitis, the frequency of diabetes was 7.5%. At 10 years and 20 years after diagnosis, it was 28% and 52%, respectively [70]. A similar trend in the incidence of diabetes associated with chronic pancreatitis was reported in another study conducted in Japan which included 656 participants. In this study, 10% of chronic pancreatitis patients developed diabetes at the onset of the study. After ten years of follow-up, the frequency of diabetes had increased dramatically to 50%; after 25 years, it was 83% [37]. While there is paucity of data on pancreatogenic diabetes mellitus among HIV-infected individuals, data from the general population can provide insight on the severity and burden of the disease. Pancreatogenic diabetes mellitus has been described as a function of inflammation-induced damage of pancreatic cells [71], caused by infections and toxic substances, such as HIV and alcohol. The link between diabetes and HIV is well-established. A large retrospective cohort study of 199,707 PLWH without history of diabetes was conducted in Thailand between 2007 and 2013. At the end of the study period, 8383 participants had developed diabetes [72]. In another population-based cohort study conducted in South Carolina using the Medicaid database, the incidence of diabetes in HIV-infected individuals was found to be higher than that of non-infected participants in a 1:1 matched case design [73]. Centers for Disease Control and Prevention (CDC) The National Health and Nutrition Examination Survey (NHANES) data, explored by Hernandez-Romieu et al., revealed a 3.8% higher prevalence of diabetes mellitus in HIV-infected individuals as compared with the general population [74]. Of note: excessive alcohol intake, which is also a risk factor for pancreatogenic diabetes mellitus, occurs more frequently among PLWH [51]. Although other studies have linked pancreatitis and diabetes among PLWH to chronic exposure to HAART, this may not be a substantial reason for pancreatogenic diabetes among alcohol-abusing HIV-infected individuals. This is because the current HAART are relatively safe and numerous alternatives are available to replace any HAART linked to abnormal serum pancreatic enzymes.

## 3. HIV-Induced Pancreatitis

### 3.1. Clinical Significance

Between 1990 and 2010, pancreatic cancer ranked as the 6th most diagnosed cancer among HIV-infected individuals in San Francisco [75]. While pancreatic cancer is the end-stage disease for pancreatic dysfunction, events starting with acute pancreatitis are significant in describing disease progression. Acute pancreatitis is a well-known complication of HIV [42] with an increasing prevalence [76]. While 2% accounts for the incidence of acute pancreatitis in the general population, 40% of PLWH may present with acute pancreatitis annually [77]. Numerous studies have linked AIDS to pancreatitis. A retrospective study reported the incidence of pancreatitis in 22% of AIDS patients [78]. Another study compared pancreatic damage in AIDS patients to non-AIDS HIV-patients; the incidence of pancreatic damage was significantly higher among the AIDS patients [79]. Additionally, as observed in another study, lower cluster of differentiation 4 (CD4) count and higher viral loads were associated with pancreatitis [42]. Moreover, evidence of pancreatitis from HIV-infected pediatric patients [80,81,82] may substantiate HIV as an independent risk factor for pancreatitis, since the manifestation of other potential risk factors among this study population is minimal. While other infectious agents such as cytomegalovirus, mycoplasma, hepatotropic viruses, aspergillus, Toxoplasma and coxsackie virus are known etiologies for pancreatitis, HIV may synergize with the aforementioned pathogens to severely assault the pancreas [83]. Hence, we do not undermine the role of these pathogens in HIV-induced pancreatic damage. Although it may be difficult to understand the role of specific organisms in the pathogenesis of pancreatitis among PLWH—given that HIV-infected individuals are usually co-infected with the above-mentioned pathogens—evidence of pancreatitis from individuals with primary HIV infection may be profound in implicating HIV as an independent risk factor for pancreatitis [84,85,86,87,88,89].

### 3.2. HIV Entry into the Pancreas

HIV entry into pancreatic cells may be the initiation point for HIV-induced pancreatotoxicity. Additionally, the role of HAART needs to be recognized, as HAART is now accessible and available to the majority of PLWH. The availability of HAART has modified the natural course of HIV; in fact, HIV has evolved from a death verdict to a manageable and treatable chronic disease. Despite these outstanding benefits of HAART, numerous side effects have been documented from chronic exposure to HAART. Acute pancreatitis is one of the side effects linked to HAART. Sulfamethoxazole-trimethoprim, pentamidine and didanosine were among the earliest drugs associated with pancreatitis among PLWH [90,91,92,93]. In the HAART era, nucleotide reverse transcriptase inhibitors are strongly implicated [93,94,95]. However, findings from other studies deviate strongly from HAART-induced pancreatitis [46,96]. Moreover, Barbosa et al. compared pancreatic damage in deceased AIDS patients during the HAART era to the pre-HAART era, and found that pancreatic damage was associated with HIV and its complications rather than HAART use [97]. Furthermore, HAART targets viral replication instead of viral annihilation [98,99], allowing HIV to assume latency and inhabit potential quiescent cell reservoirs [100,101]. HIV eradication is very intricate even during consistent HAART adherence [100,102].

HIV latency in immune cells, which act as silent reservoirs, is already known. However, the role of non-immune cells as a reservoir for HIV proviruses has only recently begun to emerge. This may affect ongoing efforts towards HIV cure. Therefore, for adequacy in successful HIV eradication, therapeutic strategies exploring latent HIV eradication should include both immune and non-immune cells. This makes effort to identify the potential HIV reservoirs indispensable. While CD4+ T cells are known as prominent HIV reservoirs [103], other cells or anatomical sites are becoming notorious for harboring latent HIV proviruses. Examples include astrocytes [104,105], microglia [106], kidneys [107], lungs [108,109,110] and genitalia [111]. While some key organs (e.g., liver) did not previously qualify as HIV reservoirs, HIV persistence in the liver after years of HAART adherence has been shown [112,113,114,115]. Additionally, while macrophages were commonly known to harbor HIV in the liver, evidence has emerged that sheds light on the role of hepatocytes as a gateway for HIV into the liver. Studies by Ganesan et al. recently supported HIV entry into hepatocytes [116], while Kong et al. showed low level replication of HIV in hepatocytes [117]. Thus, hepatocytes, while not acting as the real HIV-permissive cells, do contribute to HIV persistence in the liver.

There is evidence from clinical studies that show an HIV presence in the pancreases of PLWH. A postmortem analysis of 109 AIDS patients and 38 controls carried out within 6 h of death revealed HIV proteins (p24) in the pancreatic cells of 24 of the AIDS patients. Other opportunistic pathogens, such as pneumocystis carinii, Toxoplasma and cytomegalovirus were also reported. A correlation was found between AIDS and features of pancreatic acinar damage including decreased zymogen granules, adverse nuclear changes, atrophy, steatosis, inflammation, hemorrhage, edema and fibrosis [118]. Another study reported pancreatic abnormalities from histological examination of 113 AIDS patients. Findings from this study revealed necrotic tissue damage linked to HIV infection [119].

To confirm HIV entry into pancreatic acinar cells, we recently exposed HIV-1_ADA_ at multiplicity of infections (MOIs) ranging between 0.085 and 0.34 to SW1990 cells, a pancreatic cancer cell line. HIV gag RNA correlating with the MOIs of HIV was observed (in our unpublished observations). Although the mechanisms for HIV entry into pancreatic acinar cells have not been identified, intensive studies have been conducted on HIV entry into other non-immune cells. Meanwhile, non-immune cells are CD4 negative; therefore, the mechanisms of HIV entry into non-immune cells are CD4-independent. While human mannose receptor was identified as the HIV entry for astrocytes [120], both C-C chemokine receptor type 5 (CCR5) and CXC chemokine receptor type-4 (CXCR4) were implicated for HIV entry into renal parenchymal calls [121]. Although only CXCR4 was shown to allow HIV entry into cardiomyocytes [122,123], both CCR5- and CCR4-dependent HIV entry into hepatocytes has been suggested [117]. While no evidence is available for HIV entry receptor into pancreatic acinar cells, expressions of CCR5 have been reported on pancreatic tissues [124,125]. Although CCR5 expressed on pancreatic acinar cells play a significant role in the progression of pancreatic cancer, CCR5 have also been shown on cells of nonmalignant pancreatic tissues [126]. Furthermore, pancreatic stellate cells were shown to express CXCR4 [127].

To further determine if HIV entry into pancreatic cells is mediated by CCR5, we blocked CCR5 on SW1990 cells with a pharmacological CCR5 inhibitor (maraviroc) and measured HIV gag RNA using RT-PCR. While HIV gag RNA was detected after exposure of SW1990 to HIV, maraviroc treatment blocked HIV RNA expression (in unpublished data). CCR5 is also known as a potential receptor candidate for entry of other viruses, such as cytomegalovirus, known to target both exocrine and endocrine pancreatic cells [128]. From these, we may assume that CCR5 is the HIV entry receptor for HIV into pancreatic acinar cells.

### 3.3. HIV-Induced Damage in Acinar Cells

While we have evidence to suggest that HIV entry into pancreatic acinar cells occurs and that this may be mediated by CCR5, no mechanisms of HIV-induced pancreatitis are disclosed. However, we can make inferences from other similar nonimmune cellular systems to predict HIV-induced pathology in pancreatic acinar cells. One of the key observations commonly reported in other nonimmune cells in the context of HIV is replication restriction after HIV entry. For example, astrocytes were shown in an in vitro study to restrict HIV replication via the T-cell factor 4, which is a downstream effector of the Wnt pathway [129]. Brack–Werner also reported nonproductive replication of HIV in astrocytes [130].

Apparently, astrocytes are not the only cells shown to restrict HIV replication. Cardiomyocytes, which allow HIV entry, have demonstrated abortive HIV replication [123]. Additionally, hepatocytes were shown recently to demonstrate similar abortive HIV replication [116]. These nonimmune cells vary and may have different mechanisms mediating the restriction of HIV replication. The endpoint of HIV-containing cells in all the reviewed studies was apoptosis. While the observed abortive HIV replication was strongly linked to apoptosis, the involved mechanisms were not clear. Given that Ganesan et al. showed that HIV-exposed hepatocytes expressed HIV gag RNA p24, low reverse transcriptase activity and low total DNA with no integrated DNA [116], it may be presumed that apoptosis was triggered when the viral genome integrated with the host DNA. However, this has never been reported. It can be tested by investigating integrated HIV DNA in apoptotic cells. This is fundamental because if the replication-competent HIV particle is present in apoptotic cells, it may become a vehicle for effective HIV spread within the organ. Looking at this from another perspective, our group recently reported abortive replication and apoptosis of HIV-containing hepatocytes. This seems beneficial because it provides an avenue for HIV clearance from the organ, but ends up becoming detrimental because HIV-containing apoptotic cells activated hepatic stellate cells when they were removed [116]. While the mechanisms of HIV-induced apoptosis are under-investigated, HIV proteins are mostly implicated in cell death. Evidence from both in vitro and in vivo study in brain cells showed significant cell death after exposure to HIV envelope proteins (gp120 and gp160) even at a very low concentration of 1ng/mL [131]. Also, our group demonstrated the potential toxic effects of p24 on hepatocytes [116]. Although we did not directly measure the toxicity of p24 in hepatocytes, we observed a correlation between p24 and reactive oxygen species (ROS), which consequently resulted in apoptosis induced by activation of oxidative stress. These observations were made in hepatocytes; the mechanisms in pancreatic acinar cells may differ slightly. In fact, while HIV-induced acinar death may be explained by multiple mechanisms, the most prevalent mechanism revolves around endoplasmic reticulum (ER) and oxidative stress.

Since acinar cells are effective secretory cells for digestive enzymes, ER activity in acinar cells becomes paramount for enzyme production and folding [132]. While protein synthesis in the ER may be crucial, proteins only become functional when properly folded to their native conformation [133]. This emphasizes the importance of ER protein folding. Misfolded proteins which are not properly refolded are subjected to ER-associated protein degradation (ERAD), a pathway targeting the misfolded proteins from ER for ubiquitination and proteasomal degradation in cytosol. ER stress sensors trigger unfolded protein response (UPR), resulting in the regulation of molecular chaperones and folding enzymes to increase ER protein folding capacity. At least three UPR have been identified, e.g., inositol-requiring protein 1 (IRE1), protein kinase RNA-like ER kinase (PERK) and activating transcription factor 6 (ATF6) [134]. Although UPR is supposed to restore ER homeostasis and promote cell survival and adaptation, it is not the case for HIV. ER stress and UPR are induced by viral infections, including HIV, and prolonged ER stress may lead to apoptosis or other types of cell death [135]. In astrocytes, HIV induces UPR activation and finally upregulates such genes as BiP and CHOP [136]. It is not clear whether the same happens in acinar cells, which can also be unproductively HIV-infected. Another study on astrocytes revealed that HIV-induced ER stress was mediated by HIV-induced inflammatory cytokines. In this study, HIV-induced IL-1β was potent enough to activate all the UPR, leading to ER stress [137]. While this mechanism was observed in HIV-infected astrocytes, it might also be the case for pancreatic acinar cells, given that acinar cells are susceptible to HIV-induced inflammation [88]. The comparisons between astrocytes and pancreatic acinar cells are legitimate, since HIV infection in both these cell types is not productive. While HIV-induced inflammasome was implicated in the aforementioned study, another study utilizing astrocytes indicated gp120 (HIV envelope protein) as the trigger for ER stress. Based on the latter study, gp120 upregulated ER stress markers such as phosphorylated JNK, XBP1 splicing and AP-1, which ultimately induced caspase-3-dependent cell death [138]. HIV-triggered ER stress may be induced by other HIV proteins, such as HIV Tat. A direct induction of UPR leading to ER stress by HIV Tat has been reported [139]. A more accurate assumption, predicting the mechanism of HIV-induced ER stress in pancreatic acinar cells, was observed in the pathogenesis of Coxsackievirus, which is a pancreatotropic single stranded RNA virus. Colli et al. observed the activation of one of the UPRs, which simultaneously mediated ER stress and induced the replication of Coxsackievirus [140]. The exact mechanisms describing these events included the activation of IRE1, causing the elevation of spliced XBP1—an important marker for ER stress [141]. Another effect of Coxsackievirus-induced IRE1 is JNK1 activation, required for Coxsackievirus replication in pancreatic cells. In essence, Coxsackievirus in pancreatic cells induced ER stress and its replication. While we perceive strongly that HIV—another RNA virus—will induce similar ER stress, we may not be confident about the ability of HIV to replicate completely using this same mechanism, given that all investigated nonimmune cells mentioned in this review had abortive HIV replication [116].

The ultimate outcome of ER stress is cell death through apoptosis or necrosis; however, the prevailing cell death mechanism has not been clearly elucidated. While the pro-apoptotic functions of IRE1 have been identified through the TRAF2 and JNK pathway [142], cellular necrosis was also reported through the TRAF2-JNK pathway in the context of ER stress [143]. Indeed, many studies have preferentially reported apoptosis as the predominant ER stress-induced cell death [144,145,146], but other types of cell death triggered by ER stress are possible. To elucidate the effect of ER stress on various types of cell death, the dual functions of UPR on pro-survival [147] and pro-apoptotic proteins should be compared [148]. While these two functions are contrasting, cells may undergo apoptosis or not, depending on the degree of ER stress [145]. During mild ER stress, PERK participates actively to maintain cellular homeostasis for enhancing cell survival; however, when stress is elevated, the activation of PERK induces activating transcription factor 4 (ATF4), a component of PERK, for the inducement of apoptosis [149]. Similarly, ATF6 activates apoptosis. Although the involved mechanism has not been clearly elucidated, evidence of ATF6-induced apoptosis by mediating WW Domain Binding Protein 1 has been reported [150]. Furthermore, ER stress-induced pyroptosis has been also observed. As is known, pyroptosis is a caspase-1-mediated cell death, characterized mainly by inflammation [151]. It is important to pinpoint pyroptosis as an example of ER stress-induced cell death in HIV-induced damage of pancreatic acinar cells, given that HIV infection mediates inflammation in the pancreas. In addition, ER stress-induced caspase-1 overstimulation and consequent pyroptosis has been shown in hepatocytes [152], as has ER stress-induced liver injury mediated by IL-1β [153].

HIV-induced oxidative stress can also cause cell death. For example, glutathione depletion was observed in many HIV-infected systems [154,155,156]. Increased oxidative stress indicators, such as malondialdehyde [157,158,159], oxidized DNA [160] and 4-hydroxynonenal [161] were detected in tissues of HIV-infected individuals. Moreover, Brundu et al. observed glutathione depletion in the pancreas of mice infected with murine leukemic virus (MLV), which causes AIDS in mice [162]. This was linked to the induction of pancreatitis-like injury in AIDS-infected mice [163]. HIV proteins are likewise the active trigger of oxidative stress [164,165,166]. The mechanisms of HIV-induced oxidative stress are linked to the mitochondrion [167], which may mediate cell death [168,169]. While HIV in other nonimmune cells generates ROS to induce cell death by apoptosis, the mechanism of ROS-induced cell death in pancreatic acinar cells may include necrosis [154], which is frequently linked to pancreatitis [42,170]. Even though apoptosis and necrosis may occur simultaneously, it is possible that apoptosis in some instances may precede necrosis. A study revealed that infiltration of inflammatory cells triggered secondary necrosis in apoptotic cells [158]. Both necrosis and apoptosis of acinar cells is triggered by mitochondria membrane permeabilization, mediated by HIV-induced ROS [171]. In addition, infiltration of T helper cells to HIV-containing pancreatic acinar cells may also mediate acinar death. For example: CCR3+ T helper 1-type CD4+ cells were shown to infiltrate MLV-containing pancreatic acinar cells due to the expression of CXCL10 [163]. CXCL10 have been shown to induce apoptosis in pancreatic acinar cells [172]. This suggests that HIV-induced pancreatitis may be an autoimmune pancreatitis. This is supported by studies on case reports of diagnosed autoimmune pancreatitis of HIV-infected individuals [88,173].

**Figure 1:**
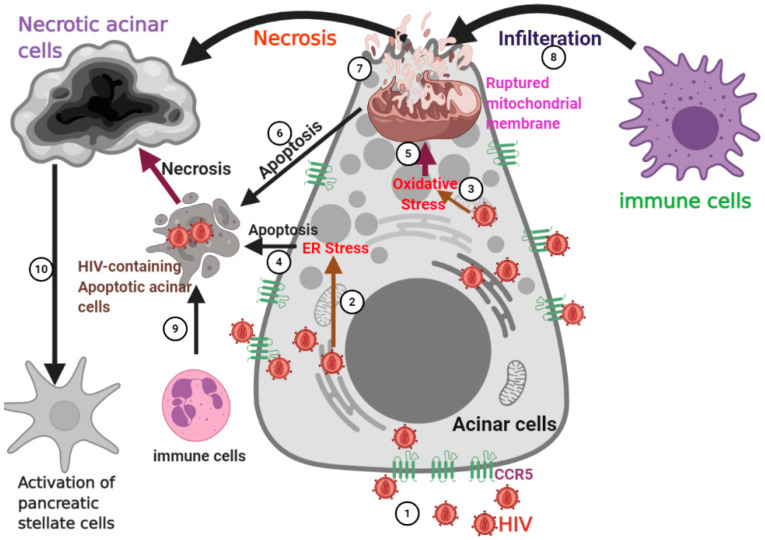
An HIV-exposed pancreas. Visual depiction of the pathogenesis of HIV-induced pancreatitis. We hypothesize that HIV undergoes some events to mediate its toxicity on pancreatic acinar cells and these include: (1) HIV entry via C-C chemokine receptor type 5 (CCR5) receptors which are adequately expressed on acinar cells; (2) HIV proteins triggering ER stress in the ER; (3) HIV proteins triggering oxidative stress in mitochondria; (4) ER stress triggering apoptosis; (5) oxidative stress triggering mitochondrial membrane rupture; (6) Ruptured mitochondrial membrane triggered apoptosis or (7) necrosis; (8) infiltrating immune cells necrotizing HIV-containing acinar cells; (9) infiltrating immune cells necrotizing HIV-containing apoptotic acinar cells; (10) necrotic acinar cells activating the pancreatic stellate cells, leading to pancreatic inflammation and fibrosis.

The pathogenesis of HIV-induced pancreatitis is beyond just acinar necrosis because, after acinar necrosis, pancreatic stellate cells become activated. The activation of pancreatic stellate cells after acinar injury or death is a well-known concept; however, the actual type of cell death that activates pancreatic stellate cells has not been well established. Some in vivo studies reported the progression of pancreatitis with increased necrosis, while apoptosis played a protective role [174,175]. The crosstalk initiated by acinar necrotic cells is intended to activate pancreatic stellate cells for the release of an extracellular matrix, to maintain tissue architecture altered during pancreatic acinar necrosis. This was previously demonstrated in the co-culture of acinar and pancreatic stellate cells, where activation of nuclear factor kappa-light-chain-enhancer of activated B cells (NFkB) and acinar necrosis was observed—with a concomitant increase in the extracellular matrix protein expression by pancreatic stellate cells [176]. While we are interested in exploring HIV-induced pancreatic acinar necrosis as the driver of the activation of pancreatic stellate cells, it is important to elucidate the known signals for pancreatic stellate cells. Evidence from in vivo studies has revealed that pancreatic stellate cells are activated by the following signals: platelet derived growth factors (PDGF), transforming growth factor beta (TGFβ), Tumor necrotic factors (TNFα), reactive oxygen species [177,178,179,180,181], IL-1, IL-6, IL-10 [182] and angiotensin II [183]. These signals upregulate fibrogenesis by producing substantial amount of extracellular matrix and collagen, leading to the progression of pancreatic damage. All these mechanisms are summarized in Figure 1.

## 4. Alcohol Potentiates HIV-Induced Pancreatitis

### 4.1. Significance

Approximately 14.1 million adult Americans reported AUD in 2019, with 95,000 deaths linked to alcohol abuse annually. Moreover, excessive use of alcohol deprives the US economy of approximately $250 billion annually, a cost which includes loss of workplace productivity, collision or automobile crashes, elevated criminal activities and healthcare [184]. Furthermore, alcohol has been associated with many morbidities, either as a risk factor or as a factor potentiating disease progression. For example: alcohol is a recognized risk factor for HIV infection and transmission [185,186]. Alcohol is also known to interfere with adherence to HAART required for virologic suppression [187,188,189,190,191,192,193,194,195]. Consequently, numerous organs in the body become exposed to the potential toxic effects of unsuppressed or rebound HIV.

We focused on the impact of alcohol on HIV-exposed pancreatic acinar cells. Just like other organs, the pancreas is massively exposed to HIV in alcohol-abusing HIV-infected individuals because of alcohol-induced failed virologic suppression or viremic rebound. This is just a broad description of the role of alcohol in HIV-exposed pancreatitis; in this review, we will provide some detail concerning the mechanistic explanation of how alcohol potentiates HIV-induced pancreatitis. Years of rigorous research on pancreatitis have shifted attention from the previously acclaimed sphincteric and pancreatic stone protein theories to pancreatic secretory cells. Currently, the action of alcohol on secretory cells is highly implicated for pancreatitis. While epidemiological studies have associated alcohol to pancreatitis [196,197,198,199] and experimental studies have demonstrated how alcohol and its metabolites induce pancreatic damage by premature activation of digestive enzymes [200,201], the role of ethanol for potentiating HIV-induced pancreatic damage is the focus of this review.

### 4.2. Pancreatic alcohol metabolism

First, we need to update our understanding on the ethanol-metabolizing tendencies of pancreatic cells. Both acinar cells and pancreatic stellate cells are known to metabolize ethanol. Previously, Norton I. demonstrated ethanol-induced cytochrome P4502E1 (CYP2E1) in rats’ pancreatic tissues, which have similar CYP2E1 expression patterns as liver cells exposed to ethanol [202]. While Norton I. demonstrated CYP2E1 only in rats’ tissues, the presence of CYP2E1 in the human pancreas was verified in another study [203]. CYP2E1 is not the only alcohol-metabolizing enzyme observed in the pancreas, as alcohol dehydrogenase (ADH), another known alcohol-metabolizing enzyme, has also been reported [204].

To evaluate the ADH polymorph expressed by pancreatic acinar cells, we exposed SW1990 cells to 4-methyl pyrazole (4-MP), an ADH1-specific inhibitor. We observed a significant downregulation of ethanol-induced ADH by 4-MP (unpublished observations). This suggests that pancreatic acinar cells may be metabolizing ethanol by ADH1. More recently, genetic studies also linked ADH1B*2 to pancreatitis [205]. Another study using human tissues observed expression of ADH1 in human pancreatic tissues [206]. Evidence of ethanol metabolite-induced pancreatotoxicity was shown by measuring malondialdehyde in ethanol-fed rats [207]. Malondialdehyde, in the context of ethanol exposure, is an indicator of acetaldehyde release and the lipid peroxidation process. This confirms the involvement of ethanol metabolites in pancreatitis. While the pancreas may be linked to oxidative alcohol metabolism, evidence of non-oxidative alcohol metabolism in the pancreas also exists [208]. In fact, substantial amounts of non-oxidative metabolites such as fatty acid ethyl ester (FAEE) in pancreatic acinar cells have been reported [209]. However, ethanol oxidative metabolism in the pancreas is higher than non-oxidative metabolism [210].

### 4.3. Alcoholic Pancreatitis

Approximately one out of four cases of pancreatitis is due to chronic alcohol consumption [211]. While alcoholic pancreatitis has been intensely described, the mechanisms of the combined effects of HIV and alcohol remain unexplored. As we attempt to understand how alcohol potentiates HIV-induced pancreatitis, it is refreshing to briefly comment on alcoholic pancreatitis. Given that alcohol metabolism in the pancreas occurs oxidatively and non-oxidatively, alcohol metabolites play a vital role in the pathogenesis of alcoholic pancreatitis. Meanwhile, sustained elevation of free calcium in acinar cytosol is known to mediate premature activation of zymogen, which triggers acinar injury [212,213,214]. The role of calcium in zymogen premature activation cannot be overemphasized. The pharmacological blockade of calcium channels was shown to completely prevent acinar cell injury even in the presence of alcohol [215]. Also, the alcohol non-oxidative metabolite FAEE was shown to participate in the upregulation of cytosolic calcium [216]. FAEE involvement in acinar injury is not limited to the disruption of calcium homeostasis; FAEE was also shown to weaken the membranes of lysosomes and zymogen granules [217,218], which also led to the premature activation of zymogen. This may occur either by FAEE-induced rupture of zymogen granule membranes or by activation of zymogen by lysosomal hydrolases leaked from FAEE-induced ruptured lysosomes [219].

**Figure 2:**
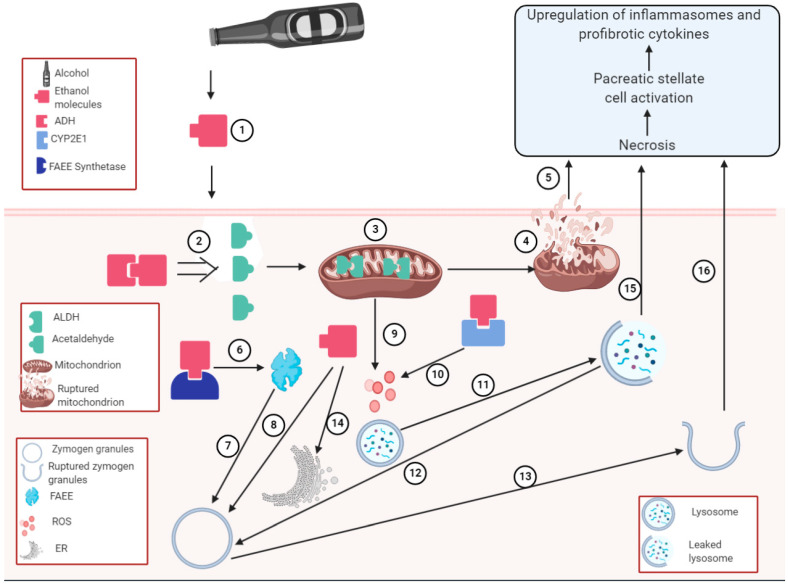
Alcoholic pancreatitis: Visual depiction of the mechanisms of alcohol-induced pancreatitis: We observed that the mechanisms of alcohol-induced pancreatitis include:(1) Exposure of cell to alcohol molecules; (2) oxidative metabolism of alcohol in the presence of alcohol dehydrogenase (ADH) to yield reactive toxic metabolite, acetaldehyde; (3) detoxification of acetaldehyde in the mitochondrion by aldehyde dehydrogenase (ALDH); (4) Oxidative stress-triggered mitochondrion membrane rupture; (5) Mitochondrion membrane rupture leading to necrosis; (6) Alcohol undergoing nonoxidative metabolism to form fatty acid ethyl esters (FAEE); (7) FAEE weakening zymogen granule membranes; (8) unmetabolized alcohol directly weakening zymogen granule membranes; (9) mitochondrion releasing ROS; (10) ethanol metabolism by CYP2E1 releasing ROS; (11) ROS rupturing lysosome membrane; (12) released lysosome hydrolases from ruptured lysosome weakening the zymogen granule membranes; (13) Zymogen granule membrane rupture and activation; (14) Ethanol upregulating UPR with no ER stress observed; (15) Ruptured lysosomes inducing necrosis; (16) Ruptured zymogen granules inducing necrosis.

FAEE is not the only ethanol metabolite known for adverse effects on acinar cells. Acetaldehyde, an alcohol oxidative metabolite, may also trigger acinar cell injury by inhibiting amylase secretion [220]. Moreover, acetaldehyde and ROS induce acinar cell injury when they undergo lipid peroxidation with lysosome and zymogen granule membranes [221]. In addition to the oxidative stress induced by acetaldehyde, alcohol was observed to increase unfolded protein response (UPR). Meanwhile, when UPR induction occurs adequately, it protects the cell and maintains cellular homeostasis. However, over-activated or prolonged UPR signaling experienced during chronic alcohol consumption may trigger ER stress [222]. Therefore, during chronic alcohol exposure, ER stress may develop in pancreatic acinar cells. Unlike other alcohol metabolizing cells, such as hepatocytes, which are injured by the induced ER stress, [223] XBP1 in acinar cells mediates the attenuation of alcohol-induced ER stress. This may be related to the fact that the ethanol-metabolizing capacity of liver cells far exceeds that of pancreatic cells and thus, the levels of oxidative and ER stresses are low in the pancreas when compared with liver cells. These stresses may not result in alcohol-induced pancreatitis [224,225] and have been considered a physiologic adaptive response for ethanol-induced pancreatitis. However, a “second hit” such as HIV may trigger ER stress [226]. Furthermore, alcohol induces the missorting of cathepsin B in such a way that it colocalizes with zymogen granules, leading to premature activation of zymogen and acinar cell injury [204,227]. While the premature activation of zymogen by lysosomal hydrolases has been established, alcohol may increase intracellular production of lysosomal hydrolases and zymogen granules, which increases the likelihood for untimely zymogen activation [228,229,230]. Moreover, alcohol may mediate acinar injury by impairing zymogen secretion, leading to accumulation of zymogen [231,232]. Decrease in the stability of zymogen granules and lysosomes due to alcohol exposure have also been reported [200,233]. The details of the mechanisms of alcohol pancreatitis are shown in Figure 2.

### 4.4. Proposed Mechanisms for the Role of Alcohol in HIV-Induced Pancreatitis

Given that HIV entry and ethanol metabolism are events that potentially occur in pancreatic cells, the next valid question is: how does ethanol (or its metabolites) affect HIV-induced pathogenesis in pancreatic cells? The impetus to study the combined effects of alcohol and HIV on pancreatic acinar cells was drawn from the following: first, the elevated prevalence of alcohol use disorder among HIV-infected individuals [234]; second, the elevated risk of pancreatitis among alcohol abusing individuals [235]; third, the fact that pancreatitis is a common occurrence among PLWH [173]. It suffices to say that, while alcohol consumption by HIV patients increases the risk of pancreatitis, HIV infection of acinar cells may be required for the manifestation of the disease. Although there is paucity of literature on studies highlighting the role of alcohol in potentiating HIV-induced pancreatitis, we relied on descriptions from other similar cellular systems to explain these mechanisms. We started by proposing alcohol-induced CCR5 modification as a possible mechanism for potentiating HIV-induced pancreatitis. It was previously shown in an in vitro study that the entry of HIV into human blood monocyte-derived macrophages was enhanced by ethanol treatment administered in a dose-dependent manner [236]. Additionally, increased CCR5 expression was shown in the liver of ethanol-fed mice [237]. Another study demonstrated the alcohol-induced elevation of CCR5 on peripheral blood lymphocytes [238]. As alcohol-induced CCR5 upregulations were observed in other cells, we were tempted to assume similar alcoholic upregulation of CCR5 for pancreatic acinar cells.

**Figure 3:**
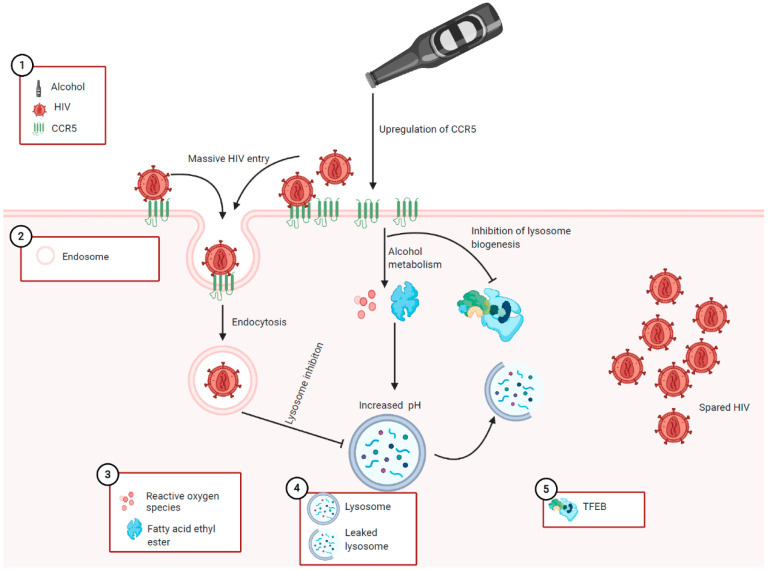
Proposed mechanisms of HIV-induced pancreatitis potentiated by alcohol: Visual illustration of the proposed mechanisms explaining how alcohol and its metabolites potentiates HIV-induced pancreatic damage. The mechanistic steps include: (1) at the surface of the acinar cell membrane, ethanol or its metabolites upregulating CCR5, leading to massive HIV entry into acinar cells; (2) HIV becomes internalized by the endosome and is fated for degradation by the pH-dependent lysosome; (3) alcohol becomes metabolized oxidatively and non-oxidatively to yield ROS and fatty acid ethyl esters (FAEE), respectively; (4) the lysosome becomes impaired due to alcohol-induced pH elevation or disruption of lysosome membrane by ROS and FAEE; (5) persistent lysosome damage due to alcohol-induced inhibition of lysosome biogenesis. The overall effects of these mechanisms lead to the accumulation and persistence of HIV, which should have been degraded by lysosomes; hence, the accumulated HIV induces the damage highlighted in Figure 1.

While HIV binds to the membrane of target cells by CCR5, viral internalization is achieved by endocytosis [239,240,241]. In fact, this may partly explain the nonproductive HIV replication commonly observed in nonimmune cells, given that internalized HIV is fated for degradation by pH-dependent lysosome [242]. However, when the lysosome becomes impaired by elevated pH, HIV accumulates in the cells. Fredericksen et al. previously observed HIV accumulation in Human 293T cells and HeLa Magi cells after increasing lysosomal pH with bafilomycin [243]. Also, alcohol was shown to be able to increase lysosome pH just like bafilomycin. This was demonstrated when Kharbanda et al. exposed rats to ethanol. A 0.2 unit increase of lysosomal pH, which was significant enough to suppress protein degradation, was observed. This effect was higher and prolonged in rats with chronic ethanol exposure [244]. Similarly, alcohol-induced lysosome dysfunction has been demonstrated in liver tissues [245,246,247,248,249]. In view of this, we recently demonstrated HIV accumulation in hepatocytes with alcohol-impaired lysosomes [116]. No studies, to our knowledge, have observed alcohol-induced HIV accumulation in pancreatic acinar cells, and we were reluctant to make inferences from other cell systems. However, we became insistent when we observed similarities between the patterns of alcohol-induced lysosome damage in other nonimmune and acinar cells (described in Section 4.3). A detailed description of the proposed mechanism by which alcohol potentiates HIV-induced pancreatitis is fully described in Figure 3.

The ultimate outcome of pancreatic acinar cells exposed to both HIV and alcohol is cell death, mediated by alcohol-induced HIV accumulation. While apoptosis is commonly linked to HIV-induced cell death, this may not be completely accurate for the pancreas. In HIV-infected CD4+Lymphocytes, only 5% were shown to account for apoptosis; the remaining 95%, which did not support productive HIV replication, died by pyroptosis [250]. Moreover, in HIV-infected monocytoid and T-lymphoblastoid cells, only 12% of HIV-induced cell death was due to apoptosis. Necrosis accounted for the remaining 88%, accompanied by some intracellular changes such as ER and mitochondrial dilation [251]. While the above mentioned mechanisms were illustrative for HIV-induced cell death in immune cells, apoptosis was predominantly observed in HIV-expressing nonimmune cells such as hepatocytes [116] and cardiomyocytes [123]. However, these studies may have potentially missed HIV-induced necrosis, given that necrosis was never measured as a mechanism of HIV-induced cell death. While HIV by itself may have provided some toxicity, as described in Section 3.3, our intent here was to describe how alcohol-induced HIV accumulation in pancreatic acinar cells may trigger a more prominent toxicity in the cells. Therefore, in future studies, we will lean towards necrosis as the predominant cell death mechanism in acinar cells exposed to both HIV and alcohol—since pancreatitis is primarily mediated by necrosis [252].

## 5. Potential Therapeutic Strategies for Alcohol and HIV-Induced Tissue Damage: A Reflection for HIV-Induced Pancreatitis Potentiated by Alcohol

While the current HAART is efficient at restricting viral replication, it may not be adequate for resolving organ damage in nonimmune systems. This is because the mechanism of HIV and alcohol-induced toxicity in nonimmune cells is independent of viral replication. As a result, an effective therapeutic regimen required to ameliorate the adverse effects of HIV and alcohol is required to augment HAART. So far, from evidence garnered in this review, we know that HIV entry into many nonimmune cells is CCR5-dependent, which is triggered by ethanol metabolites, leading to intracellular HIV accumulation. The two major mechanisms identified to explain HIV accumulation in ethanol-treated nonimmune cells are CCR5 upregulation and lysosome suppression. HIV proteins from accumulated HIV perpetrate adverse effects, such as oxidative and ER stress, which leads to cell death that in turn leads to fibrosis in nonimmune organs containing fibroblasts.

Based on this understanding, therapeutic regimens should target suppression of HIV entry, resuscitation of lysosome functions, suppression of cell death, and finally, suppression of pancreatic stellate cell activation. As we explore available therapeutic regimens for the above listed therapeutic targets, it is important to deliberate on why inhibiting only cell death may not be efficient as a therapeutic strategy, even though cell death is the axis for HIV and ethanol-induced organ failure. This is because inhibition of cell death may increase HIV persistence in tissues, which may further be a source of rebound viremia when HAART use is interrupted [253,254]. Moreover, we observed in our laboratory that inhibition of apoptosis of HIV-infected hepatocytes with pan-caspase inhibitors significantly upregulated HIV gag RNA and p24 [116]. Therefore, an effective therapeutic regimen for HIV and alcohol- induced organ damage must be comprehensive.

Different types of HIV entry inhibitors exist. Given that nonimmune cells are CD4- negative, our focus will be inhibitors for HIV coreceptors and HIV envelope proteins. Very recently (July 2020), Fostemsavir was United States Food and Drug Administration (FDA)-approved for use by HIV patients. The active moiety of Fostemsavir is Temsavir, which interacts with gp120 and inhibits it from binding to CCR5 on target cells [255]. Hence, Temsavir may be efficient for inhibiting HIV entry into CCR5-expressing cells such as pancreatic acinar cells. Maraviroc is another HIV entry inhibitor approved by the FDA. It prevents HIV entry by acting as a CCR5 antagonist [256]. Leronlimab is another HIV entry inhibitor which targets CCR5 as well. While the FDA recently granted a fast-track designation for Leronlimab to augment HAART, it is still predominantly in the investigative stage in other countries. Although the potentials of Leronlimab have been demonstrated in other critical conditions, such as breast cancer, here, we are focused on HIV. Leronlimab blocks CCR5 and prevents the interaction of HIV surface proteins with CCR5 [257]. While the potency and efficacy of HIV entry inhibitors have been established by different clinical trials, no studies highlighted their specific effects on HIV and alcohol-induced organ failure. Although they may potently ameliorate HIV toxicity, it is feared that there may be other mechanisms beyond coreceptors for HIV entry into these organs. Moreover, other nonclassical HIV entry mechanisms for numerous nonimmune cells are still being studied. Targeting HIV entry as a therapeutic regimen will only be successful if all potential HIV entry mechanisms are adequately considered [240].

Resuscitation of impaired lysosome function is another opportunity for therapeutic intervention in HIV and alcohol-induced organ damage. While we seek to identify potent regimens to restore lysosome damage, we must first agree on the mechanisms that impair lysosomes in the presence of alcohol and HIV. Given that lysosome leakage triggered by oxidative stress is implicated as the mechanism for alcohol-induced lysosome damage [258], treatment with antioxidants may restore lysosome function. Recently, in our laboratory, we pretreated hepatocytes with N-acetyl cysteine (NAC), a known antioxidant, and observed a significant restoration of cathepsin B and L activities, which drastically suppress HIV gag RNA even after exposure to ethanol metabolites and HIV (unpublished observations). This indicates that NAC prevented lysosome membrane permeabilization by scavenging ROS released by ethanol metabolites and improved HIV degradation. Other studies have confirmed our findings [259,260]. While lysosome leakage is one way to explain alcohol-induced lysosome dysfunction, the modification of lysosome biogenesis is another [261,262], and it will be of immense value for resuscitation of impaired lysosome function.

Another suitable target for therapeutic purposes is stellate cells or fibroblasts of non-immune organs. Antifibrotic and anti-inflammatory agents may be efficient for ameliorating HIV- and ethanol-induced toxicity. As we have shown, an example of such an agent is obeticholic acid. Obeticholic acid is an FDA-approved drug for primary biliary cholangitis treatment. As an antifibrotic and anti-inflammatory agent, it binds to the farnesoid-X receptor (FXR) to mediate its effects. We demonstrated its ability to restore lysosome function, decrease HIV accumulation and decrease apoptosis in hepatocytes [263]. In fact, many nonimmune cells express FXR, including pancreatic cells, and thus obeticholic acid may be a suitable therapeutic regimen for HIV and alcohol-induced organ failure [264,265].

To further address fibrosis in nonimmune organs, phytochemicals with anti-inflammatory and antifibrotic properties have been explored in clinical trials. For example, the antifibrotic and anti-inflammatory properties of curcumin have been observed [266,267]. Furthermore, the antifibrotic and anti-inflammatory effects of epigallocatechin gallate have also been observed. In fact, epigallocatechin gallate attenuates ethanol-mediated activation of pancreatic stellate cells [268].

Given that there are currently no established guidelines for treating pancreatitis in alcohol-abusing HIV-infected patients, the administration of potential therapy addressing the toxic effects of HIV and alcohol seems to be the most valuable therapeutic approach.

## 6. Conclusions

In this review, we explored the mechanisms of HIV- and alcohol-induced pancreatic damage. We found that HIV entry into pancreatic acinar cells may occur via CCR5, which is key in the pathogenesis of pancreatitis in HIV-infected individuals. Moreover, we found that HIV-induced toxicity in pancreatic acinar cells is mediated by oxidative and ER stress, which induces necrosis by rupturing the mitochondrial membrane. Hence, pancreatic stellate cells become activated by interacting with necrotic products, leading to the progression of pancreatic injury. On the other hand, alcohol-induced pancreatitis is mediated directly by both oxidative and nonoxidative alcohol metabolites. Alcohol-induced oxidative stress and nonoxidative metabolites are implicated in oxidative stress and rupture of zymogen granule membrane respectively. The crosstalk between leaked lysosomes and zymogen granules has been shown to induce premature activation of zymogen by lysosome hydrolases, leading to acinar injury.

While HIV and alcohol both contribute to the development of pancreatitis, the combined effects of both have not previously been reported. To explain the possible mechanisms for alcohol- and HIV-induced pancreatitis, we proposed that alcohol enhances HIV entry into acinar cells by upregulating CCR5 expression. Furthermore, alcohol metabolites block the degradation of internalized HIV proteins to trigger ER and oxidative stress for the promotion of pancreatic acinar injury and necrosis. Interactions between the necrotic products of pancreatic acinar cells activate the pancreatic stellate cells, resulting in release of inflammasomes and profibrogenic cytokines, which mediate pancreatitis. Considering HIV entry and activation of stellate cells to be the main events that lead to HIV-induced organ damage, effective therapeutic regimens for pancreatitis should block CCR5 and suppress the activation of fibroblasts after exposure to cell death products.

## Data Availability

Not applicable.

## References

[1] World Health Organization Global Health Observatory Data. https://www.who.int/gho/hiv/en/.

[2] Haynes B.F., Burton D.R., Mascola J.R. (2019). Multiple roles for HIV broadly neutralizing antibodies. Sci. Transl. Med..

[3] Trejos-Castillo E. (2019). Technology Platforms and Family Engagement for HIV/AIDS Prevention: Addressing the Needs of Minority Rural Youth. J. Adolesc. Health.

[4] Center for Disease Control and Prevention CDC Fact Sheet. https://www.cdc.gov/nchhstp/newsroom/docs/factsheets/todaysepidemic-508.pdf.

[5] Center for Disease Control and Prevention AIDS and HIV. https://www.cdc.gov/nchs/fastats/aids-hiv.htm.

[6] Palella F.J., Chmiel J.S., Moorman A.C., Holmberg S.D., Investigators H.O.S. (2002). Durability and predictors of success of highly active antiretroviral therapy for ambulatory HIV-infected patients. Aids.

[7] Johnson L.F., May M.T., Dorrington R.E., Cornell M., Boulle A., Egger M., Davies M.-A. (2017). Estimating the impact of antiretroviral treatment on adult mortality trends in South Africa: A mathematical modelling study. PLoS Med..

[8] Mocroft A., Ledergerber B., Katlama C., Kirk O., Reiss P., Monforte A.D., Knysz B., Dietrich M., Phillips A.N., Lundgren J.D. (2003). Decline in the AIDS and death rates in the EuroSIDA study: An observational study. Lancet.

[9] Yang H.-Y., Beymer M.R., Suen S.-C. (2019). Chronic Disease Onset Among People Living with HIV and AIDS in a Large Private Insurance Claims Dataset. Sci. Rep..

[10] Harrison K.M., Song R., Zhang X. (2010). Life expectancy after HIV diagnosis based on national HIV surveillance data from 25 states, United States. JAIDS J. Acquir. Immune Defic. Syndr..

[11] Teeraananchai S., Kerr S., Amin J., Ruxrungtham K., Law M. (2017). Life expectancy of HIV-positive people after starting combination antiretroviral therapy: A meta-analysis. HIV Med..

[12] Pettit A.C., Giganti M.J., Ingle S.M., May M.T., Shepherd B.E., Gill M.J., Fätkenheuer G., Abgrall S., Saag M.S., Del Amo J. (2018). Increased non-AIDS mortality among persons with AIDS-defining events after antiretroviral therapy initiation. J. Int. AIDS Soc..

[13] Prevention, HIV among People Aged 50 and over. https://www.cdc.gov/hiv/group/age/olderamericans/index.html.

[14] Escota G.V., O’Halloran J.A., Powderly W.G., Presti R.M. (2018). Understanding mechanisms to promote successful aging in persons living with HIV. Int. J. Infect. Dis..

[15] Triant V.A., Lee H., Hadigan C., Grinspoon S.K. (2007). Increased acute myocardial infarction rates and cardiovascular risk factors among patients with human immunodeficiency virus disease. J. Clin. Endocrinol. Metab..

[16] Neuhaus J., Angus B., Kowalska J.D., La Rosa A., Sampson J., Wentworth D., Mocroft A. (2010). Risk of all-cause mortality associated with non-fatal AIDS and serious non-AIDS events among adults infected with HIV. Aids.

[17] Galli L., Salpietro S., Pellicciotta G., Galliani A., Piatti P., Hasson H., Guffanti M., Gianotti N., Bigoloni A., Lazzarin A. (2012). Risk of type 2 diabetes among HIV-infected and healthy subjects in Italy. Eur. J. Epidemiol..

[18] Herrin M., Tate J.P., Akgün K.M., Butt A.A., Crothers K., Freiberg M.S., Gibert C.L., Leaf D.A., Rimland D., Rodriguez-Barradas M.C. (2016). Weight gain and incident diabetes among HIV infected-veterans initiating antiretroviral therapy compared to uninfected individuals. JAIDS J. Acquir. Immune Defic. Syndr..

[19] Willig A.L., Overton E.T. (2016). Metabolic complications and glucose metabolism in HIV infection: A review of the evidence. Curr. HIV/AIDS Rep..

[20] Schulte-Hermann K., Schalk H., Haider B., Hutterer J., Gmeinhart B., Pichler K., Brath H., Dorner T.E. (2016). Impaired lipid profile and insulin resistance in a cohort of Austrian HIV patients. J. Infect. Chemother..

[21] Noumegni S.R.N., Nansseu J.R., Ama V.J.M., Bigna J.J., Assah F.K., Guewo-Fokeng M., Leumi S., Katte J.-C., Dehayem M., Kengne A.P. (2017). Insulin resistance and associated factors among HIV-infected patients in sub-Saharan Africa: A cross sectional study from Cameroon. Lipids Health Dis..

[22] Natsag J., Erlandson K.M., Sellmeyer D.E., Haberlen S.A., Margolick J., Jacobson L.P., Palella F.J., Koletar S.L., Lake J.E., Post W.S. (2017). HIV infection is associated with increased fatty infiltration of the thigh muscle with aging independent of fat distribution. PLoS ONE.

[23] Guimarães M.M.M., Greco D.B., Moreira A.N., Guimarães N.S., Freire C.M.V., Rohlfs B.G., Machado L.J.D.C. (2018). Lipid accumulation product index in HIV-infected patients: A marker of cardiovascular risk. Braz. J. Infect. Dis..

[24] Kilbourne A., Justice A., Rabeneck L., Rodriguez-Barradas M., Weissman S. (2001). General medical and psychiatric comorbidity among HIV-infected veterans in the post-HAART era. J. Clin. Epidemiol..

[25] Sarkar S., Brown T.T. (2019). Diabetes in People Living with HIV. Endotext [Internet].

[26] Longenecker C.T., Jiang Y., Yun C.-H., Debanne S., Funderburg N.T., Lederman M.M., Storer N., Labbato D.E., Bezerra H.G., McComsey G.A. (2013). Perivascular fat, inflammation, and cardiovascular risk in HIV-infected patients on antiretroviral therapy. Int. J. Cardiol..

[27] Beires M.T., Silva-Pinto A., Santos A.C., Madureira A.J., Pereira J., Carvalho D., Sarmento A., Freitas P. (2018). Visceral adipose tissue and carotid intima-media thickness in HIV-infected patients undergoing cART: A prospective cohort study. BMC Infect. Dis..

[28] Butt A.A., McGinnis K., Rodriguez-Barradas M.C., Crystal S., Simberkoff M., Goetz M.B., Leaf D., Justice A.C. (2009). HIV infection and the risk of diabetes mellitus. Aids.

[29] Gebrie A., Tesfaye B., Gebru T., Adane F., Abie W., Sisay M. (2020). Diabetes mellitus and its associated risk factors in patients with human immunodeficiency virus on anti-retroviral therapy at referral hospitals of Northwest Ethiopia. Diabetol. Metab. Syndr..

[30] Lee Y.-K., Huang M.-Y., Hsu C.-Y., Su Y.-C. (2016). Bidirectional relationship between diabetes and acute pancreatitis: A population-based cohort study in Taiwan. Medicine.

[31] Shen H.-N., Yang C.-C., Chang Y.-H., Lu C.-L., Li C.-Y. (2015). Risk of diabetes mellitus after first-attack acute pancreatitis: A national population-based study. Am. J. Gastroenterol..

[32] Grinspoon S.K., Bilezikian J.P. (1992). HIV disease and the endocrine system. N. Engl. J. Med..

[33] Schwartz M.S., Brandt L.J. (1989). The spectrum of pancreatic disorders in patients with the acquired immune deficiency syndrome. Am. J. Gastroenterol..

[34] Cappell M.S., Marks M. (1995). Acute pancreatitis in HIV-seropositive patients: A case control study of 44 patients. Am. J. Med..

[35] Dutta S., Ting C., Lai L. (1997). Study of prevalence, severity, and etiological factors associated with acute pancreatitis in patients infected with human immunodeficiency virus. Am. J. Gastroenterol..

[36] Dassopoulos T., Ehrenpreis E.D. (1999). Acute pancreatitis in human immunodeficiency virus–infected patients: A review. Am. J. Med..

[37] Ito T., Otsuki M., Itoi T., Shimosegawa T., Funakoshi A., Shiratori K., Naruse S., Kuroda Y. (2007). The Research Committee of Intractable Diseases of the Pancreas. Pancreatic diabetes in a follow-up survey of chronic pancreatitis in Japan. J. Gastroenterol..

[38] Monroe A.K., Glesby M.J., Brown T.T. (2015). Diagnosing and managing diabetes in HIV-infected patients: Current concepts. Clin. Infect. Dis..

[39] Carr A., Cooper D.A. (2000). Adverse effects of antiretroviral therapy. Lancet.

[40] Salehian B., Bilas J., Bazargan M., Abbasian M. (2005). Prevalence and incidence of diabetes in HIV-infected minority patients on protease inhibitors. J. Natl. Med. Assoc..

[41] Kalra S., Agrawal N. (2013). Diabetes and HIV: Current understanding and future perspectives. Curr. Diabetes Rep..

[42] Dragovic G. (2013). Acute pancreatitis in HIV/AIDS patients: An issue of concern. Asian Pac. J. Trop. Biomed..

[43] Apostolova N., Blas-Garcia A., Esplugues J.V. (2011). Mitochondrial toxicity in HAART: An overview of in vitro evidence. Curr. Pharm. Des..

[44] Moore R.D., Keruly J.C., Chaisson R.E. (2001). Incidence of pancreatitis in HIV-infected patients receiving nucleoside reverse transcriptase inhibitor drugs. Aids.

[45] Dragovic G., Milic N., Jevtovic D. (2005). Incidence of acute pancreatitis and nucleoside reverse transcriptase inhibitors usage. Int. J. STD AIDS.

[46] Smith C.J., Olsen C.H., Mocroft A., Viard J.P., Staszewski S., Panos G., Staub T., Blaxhult A., Vetter N., Lundgren J.D. (2008). The role of antiretroviral therapy in the incidence of pancreatitis in HIV-positive individuals in the EuroSIDA study. Aids.

[47] Nachega J.B., Trotta M.P., Nelson M., Ammassari A. (2009). Impact of metabolic complications on antiretroviral treatment adherence: Clinical and public health implications. Curr. HIV/AIDS Rep..

[48] Chapman S.J., Woolley I.J., Visvanathan K., Korman T.M. (2007). Acute pancreatitis caused by tipranavir/ritonavir-induced hypertriglyceridaemia. Aids.

[49] Petrov M.S., Yadav D. (2019). Global epidemiology and holistic prevention of pancreatitis. Nat. Rev. Gastroenterol. Hepatol..

[50] Conigliaro J., Justice A.C., Gordon A.J., Bryant K., Alcohol V., Group B.C.R. (2006). Role of alcohol in determining human immunodeficiency virus (HIV)-relevant outcomes: A conceptual model to guide the implementation of evidence-based interventions into practice. Med. Care.

[51] Galvan F.H., Bing E.G., Fleishman J.A., London A.S., Caetano R., Burnam M.A., Longshore D., Morton S.C., Orlando M., Shapiro M. (2002). The prevalence of alcohol consumption and heavy drinking among people with HIV in the United States: Results from the HIV Cost and Services Utilization Study. J. Stud. Alcohol.

[52] Zagaria M. (2011). Acute pancreatitis: Risks, causes, and mortality in older adults. US Pharm..

[53] Testoni P.A. (2014). Acute recurrent pancreatitis: Etiopathogenesis, diagnosis and treatment. World J. Gastroenterol..

[54] Xiao A.Y., Tan M.L., Wu L.M., Asrani V.M., Windsor J.A., Yadav D., Petrov M.S. (2016). Global incidence and mortality of pancreatic diseases: A systematic review, meta-analysis, and meta-regression of population-based cohort studies. Lancet Gastroenterol. Hepatol..

[55] Kurti F., Shpata V., Kuqo A., Duni A., Roshi E., Basho J. (2015). Incidence of acute pancreatitis in Albanian population. Mater. Socio-Med..

[56] Roberts S.E., Morrison-Rees S., John A., Williams J.G., Brown T.H., Samuel D.G. (2017). The incidence and aetiology of acute pancreatitis across Europe. Pancreatology.

[57] Karimi M., Bruns A., Maisonneuve P., Lowenfels A.B. (2009). Temporal trends in incidence and severity of acute pancreatitis in Lüneburg County, Germany: A population-based study. Pancreatology.

[58] Spanier B.W.M., Dijkgraaf M.G., Bruno M.J. (2008). Trends and forecasts of hospital admissions for acute and chronic pancreatitis in the Netherlands. Eur. J. Gastroenterol. Hepatol..

[59] Stimac D., Mikolasevic I., Krznaric-Zrnic I., Radic M., Milic S. (2013). Epidemiology of acute pancreatitis in the North Adriatic Region of Croatia during the last ten years. Gastroenterol. Res. Pract..

[60] Worning H. (1994). Acute pancreatitis in Denmark. Ugeskr. Laeger.

[61] McKay C., Evans S., Sinclair M., Carter C., Imrie C. (1999). High early mortality rate from acute pancreatitis in Scotland, 1984–1995. Br. J. Surg..

[62] Méndez-Bailón M., de Miguel Yanes J.M., Jiménez-García R., Hernández-Barrera V., Pérez-Farinós N., López-de-Andrés A. (2015). National trends in incidence and outcomes of acute pancreatitis among type 2 diabetics and non-diabetics in Spain (2001–2011). Pancreatology.

[63] Jaakkola M., Nordback I. (1993). Pancreatitis in Finland between 1970 and 1989. Gut.

[64] Głuszek S., Kozieł D. (2012). Prevalence and progression of acute pancreatitis in the Świętokrzyskie Voivodeship population. Pol. Prz. Chir..

[65] Ouyang G., Pan G., Liu Q., Wu Y., Liu Z., Lu W., Li S., Zhou Z., Wen Y. (2020). The global, regional, and national burden of pancreatitis in 195 countries and territories, 1990–2017: A systematic analysis for the Global Burden of Disease Study 2017. BMC Med..

[66] Lew D., Afghani E., Pandol S. (2017). Chronic Pancreatitis: Current Status and Challenges for Prevention and Treatment. Dig Dis Sci.

[67] Yang A.L., Vadhavkar S., Singh G., Omary M.B. (2008). Epidemiology of alcohol-related liver and pancreatic disease in the United States. Arch. Intern. Med..

[68] Peery A.F., Dellon E.S., Lund J., Crockett S.D., McGowan C.E., Bulsiewicz W.J., Gangarosa L.M., Thiny M.T., Stizenberg K., Morgan D.R. (2012). Burden of gastrointestinal disease in the United States: 2012 update. Gastroenterology.

[69] Hart P.A., Bellin M.D., Andersen D.K., Bradley D., Cruz-Monserrate Z., Forsmark C.E., Goodarzi M.O., Habtezion A., Korc M., Kudva Y.C. (2016). Type 3c (pancreatogenic) diabetes mellitus secondary to chronic pancreatitis and pancreatic cancer. Lancet Gastroenterol. Hepatol..

[70] Wang W., Guo Y., Liao Z., Zou D.-W., Jin Z.-D., Zou D.-J., Jin G., Hu X.-G., Li Z.-S. (2011). Occurrence of and risk factors for diabetes mellitus in Chinese patients with chronic pancreatitis. Pancreas.

[71] Ewald N., Kaufmann C., Raspe A., Kloer H., Bretzel R., Hardt P. (2012). Prevalence of diabetes mellitus secondary to pancreatic diseases (type 3c). Diabetes/Metab. Res. Rev..

[72] Abu-El-Haija M., Hornung L., Denson L.A., Husami A., Lin T.K., Matlock K., Nathan J.D., Palermo J.J., Thompson T., Valencia C.A. (2018). Prevalence of abnormal glucose metabolism in pediatric acute, acute recurrent and chronic pancreatitis. PLoS ONE.

[73] Tripathi A., Liese A., Jerrell J., Zhang J., Rizvi A., Albrecht H., Duffus W. (2014). Incidence of diabetes mellitus in a population-based cohort of HIV-infected and non-HIV-infected persons: The impact of clinical and therapeutic factors over time. Diabet. Med..

[74] Hernandez-Romieu A.C., Garg S., Rosenberg E.S., Thompson-Paul A.M., Skarbinski J. (2017). Is diabetes prevalence higher among HIV-infected individuals compared with the general population? Evidence from MMP and NHANES 2009–2010. BMJ Open Diabetes Res. Care.

[75] San Francisco Department of Public Health HIV Epidemiology Annual Report 2017. https://www.sfdph.org/dph/files/reports/RptsHIVAIDS/AnnualReport2017-Green-20180904-Web.pdf.

[76] Peery A.F., Crockett S.D., Barritt A.S., Dellon E.S., Eluri S., Gangarosa L.M., Jensen E.T., Lund J.L., Pasricha S., Runge T. (2015). Burden of gastrointestinal, liver, and pancreatic diseases in the United States. Gastroenterology.

[77] Oliveira N.M., Ferreira F.A.Y., Yonamine R.Y., Chehter E.Z. (2014). Antiretroviral drugs and acute pancreatitis in HIV/AIDS patients: Is there any association? A literature review. Einstein (Sao Paulo).

[78] Dowell S., Holt E., Murphy F. Pancreatitis Associated with HIV infection. Proceedings of the Fifth International Conference on AIDS.

[79] Pezzilli R., Gullo L., Ricchi E., Costigliola P., Sprovieri G., Pilati G., Fontana G. (1992). Serum pancreatic enzymes in HIV-seropositive patients. Dig. Dis. Sci..

[80] Carroccio A., Fontana M., Spagnuolo M., Zuin G., Montalto G., Canani R.B., Verghi F., Di Martino D., Bastoni K., Buffardi F. (1998). Pancreatic dysfunction and its association with fat malabsorption in HIV infected children. Gut.

[81] Carroccio A., Di Prima L., Di Grigoli C., Soresi M., Farinella E., Di Martino D., Guarino A., Notarbartolo A., Montalto G. (1999). Exocrine pancreatic function and fat malabsorption in human immunodeficiency virus-infected patients. Scand. J. Gastroenterol..

[82] Bitar A., Altaf M., Sferra T.J. (2012). Acute pancreatitis: Manifestation of acute HIV infection in an adolescent. Am. J. Case Rep..

[83] Parenti D.M., Steinberg W., Kang P. (1996). Infectious causes of acute pancreatitis. Pancreas.

[84] Rizzardi G.P., Tambussi G., Lazzarin A. (1997). Acute pancreatitis during primary HIV-1 infection. N. Engl. J. Med..

[85] Mortier E., Gaba S., Mari I., Vinceneux P., Pouchot J. (2002). Acute pancreatitis during primary HIV-1 infection. Am. J. Gastroenterol..

[86] Tyner R., Turett G. (2004). Primary human immunodeficiency virus infection presenting as acute pancreatitis. South. Med. J..

[87] Paño-Pardo J.R., Alcaide M.L., Abbo L., Dickinson G. (2009). Primary HIV infection with multisystemic presentation. Int. J. Infect. Dis..

[88] Bhurwal A., Sapru S., Ramasamy D. (2018). Diffuse Pancreatic Inflammation in an HIV Infected Individual with Elevated IgG4 Levels. J. Med. Cases.

[89] Bhurwal A., Sapru S., Ramasamy D. (2016). Diffuse Pancreatic Inflammation in an HIV Patient Masquerading as Autoimmune Pancreatitis: A Case Report: 1306. Am. J. Gastroenterol..

[90] Tanowitz H.B., Simon D., Wittner M. (1992). Gastrointestinal manifestations. Med. Clin. N. Am..

[91] Schwartz M.S., Cappell M.S. (1989). Pentamidine-associated pancreatitis. Dig. Dis. Sci..

[92] Murphey S.A., Josephs A.S. (1981). Acute pancreatitis associated with pentamidine therapy. Arch. Intern. Med..

[93] Blanchard J.N., Wohlfeiler M., Canas A., King K., Lonergan J.T. (2003). Pancreatitis treated with didanosine and tenofovir disoproxil fumarate. Clin. Infect. Dis..

[94] Callens S., De Schacht C., Huyst V., Colebunders R. (2003). Pancreatitis in an HIV-infected person on a tenofovir, didanosine and stavudine containing highly active antiretroviral treatment. J. Infect..

[95] Longhurst H., Pinching A. (2001). Pancreatitis associated with hydroxyurea in combination with didanosine. BMJ.

[96] Barrios A., Negredo F., Vilaro-Rodriguez J., Domingo P., Estrada V., Labarga P., Asensi V., Morales D., Santos J., Terron J. Safety and efficacy of a QD simplification regimen. Proceedings of the 11th Conference on Retroviruses and Opportunistic Infections.

[97] Barbosa A.G., Chehter E.Z., Bacci M.R., Mader A.A., Fonseca F.L. (2013). AIDS and the pancreas in the HAART era: A cross sectional study. Int. Arch. Med..

[98] Arts E.J., Hazuda D.J. (2012). HIV-1 antiretroviral drug therapy. Cold Spring Harb. Perspect. Med..

[99] Permanyer M., Ballana E., Ruiz A., Badia R., Riveira-Munoz E., Gonzalo E., Clotet B., Esté J.A. (2012). Antiretroviral agents effectively block HIV replication after cell-to-cell transfer. J. Virol..

[100] Sengupta S., Siliciano R.F. (2018). Targeting the latent reservoir for HIV-1. Immunity.

[101] Shan L., Deng K., Gao H., Xing S., Capoferri A.A., Durand C.M., Rabi S.A., Laird G.M., Kim M., Hosmane N.N. (2017). Transcriptional reprogramming during effector-to-memory transition renders CD4+ T cells permissive for latent HIV-1 infection. Immunity.

[102] Dahabieh M.S., Battivelli E., Verdin E. (2015). Understanding HIV latency: The road to an HIV cure. Annu. Rev. Med..

[103] Chun T.-W., Engel D., Berrey M.M., Shea T., Corey L., Fauci A.S. (1998). Early establishment of a pool of latently infected, resting CD4+ T cells during primary HIV-1 infection. Proc. Natl. Acad. Sci. USA.

[104] Chauhan A. (2015). Enigma of HIV-1 latent infection in astrocytes: An in-vitro study using protein kinase C agonist as a latency reversing agent. Microbes Infect..

[105] Chauhan A., Tikoo A., Patel J., Abdullah A.M. (2014). HIV-1 endocytosis in astrocytes: A kiss of death or survival of the fittest?. Neurosci. Res..

[106] Gray L.R., Roche M., Flynn J.K., Wesselingh S.L., Gorry P.R., Churchill M.J. (2014). Is the central nervous system a reservoir of HIV-1?. Curr. Opin. HIV AIDS.

[107] Canaud G., Dejucq-Rainsford N., Avettand-Fenoël V., Viard J.-P., Anglicheau D., Bienaimé F., Muorah M., Galmiche L., Gribouval O., Noël L.-H. (2014). The kidney as a reservoir for HIV-1 after renal transplantation. J. Am. Soc. Nephrol..

[108] Devadoss D., Singh S.P., Acharya A., Do K.C., Periyasamy P., Manevski M., Mishra N., Tellez C., Ramakrishnan S., Belinsky S. (2020). Lung Bronchial Epithelial Cells are HIV Targets for Proviral Genomic Integration. bioRxiv.

[109] Costiniuk C.T., Jenabian M.A. (2014). The lungs as anatomical reservoirs of HIV infection. Rev. Med. Virol..

[110] Cribbs S.K., Lennox J., Caliendo A.M., Brown L.A., Guidot D.M. (2015). Healthy HIV-1-infected individuals on highly active antiretroviral therapy harbor HIV-1 in their alveolar macrophages. Aids Res. Hum. Retrovir..

[111] Chikwari C.D., Dringus S., Ferrand R.A. (2018). Barriers to, and emerging strategies for, HIV testing among adolescents in sub-Saharan Africa. Curr. Opin. HIV AIDS.

[112] Blackard J.T., Ma G., Martin C.M., Rouster S.D., Shata M.T., Sherman K.E. (2011). HIV variability in the liver and evidence of possible compartmentalization. Aids Res. Hum. Retrovir..

[113] Igarashi T., Brown C.R., Endo Y., Buckler-White A., Plishka R., Bischofberger N., Hirsch V., Martin M.A. (2001). Macrophage are the principal reservoir and sustain high virus loads in rhesus macaques after the depletion of CD4+ T cells by a highly pathogenic simian immunodeficiency virus/HIV type 1 chimera (SHIV): Implications for HIV-1 infections of humans. Proc. Natl. Acad. Sci. USA.

[114] Kandathil A.J., Sugawara S., Goyal A., Durand C.M., Quinn J., Sachithanandham J., Cameron A.M., Bailey J.R., Perelson A.S., Balagopal A. (2018). No recovery of replication-competent HIV-1 from human liver macrophages. J. Clin. Investig..

[115] Kandathil A., Durand C., Quinn J., Cameron A., Thomas D., Balagopal A. Liver macrophages and HIV-1 persistence. Proceedings of the Conference on Retroviruses and Opportunistic Infections (CROI).

[116] Ganesan M., New-Aaron M., Dagur R.S., Makarov E., Wang W., Kharbanda K.K., Kidambi S., Poluektova L.Y., Osna N.A. (2019). Alcohol Metabolism Potentiates HIV-Induced Hepatotoxicity: Contribution to End-Stage Liver Disease. Biomolecules.

[117] Kong L., Maya W.C., Moreno-Fernandez M.E., Ma G., Shata M.T., Sherman K.E., Chougnet C., Blackard J.T. (2012). Low-level HIV infection of hepatocytes. Virol. J..

[118] Chehter E.Z., Longo M.A., Laudanna A.A., Duarte M.I.S. (2000). Involvement of the pancreas in AIDS: A prospective study of 109 post-mortems. Aids.

[119] Bricaire F., Marche C., Zoubi D., Saimot A., Regnier B. (1988). HIV and the pancreas. Lancet.

[120] Liu Y., Liu H., Kim B.O., Gattone V.H., Li J., Nath A., Blum J., He J.J. (2004). CD4-independent infection of astrocytes by human immunodeficiency virus type 1: Requirement for the human mannose receptor. J. Virol..

[121] Eitner F., Cui Y., Hudkins K.L., Stokes M.B., Segerer S., Mack M., Lewis P.L., Abraham A.A., Schlöndorff D., Gallo G. (2000). Chemokine receptor CCR5 and CXCR4 expression in HIV-associated kidney disease. J. Am. Soc. Nephrol..

[122] Silva M., Chen F., Shannon K., Geng Y., Klitzner T., Krogstad P. (1999). Expression of CXCR4 in Human Fetal Cardiac Myocytes: A Role in HIV Related Cardiomyopathy?. Pediatric Res..

[123] de Campos W.R.L., Chirwa N., London G., Rotherham L.S., Morris L., Mayosi B.M., Khati M. (2014). HIV-1 subtype C unproductively infects human cardiomyocytes in vitro and induces apoptosis mitigated by an anti-Gp120 aptamer. PLoS ONE.

[124] Huang H., Zepp M., Georges R.B., Jarahian M., Kazemi M., Eyol E., Berger M.R. (2020). The CCR5 antagonist maraviroc causes remission of pancreatic cancer liver metastasis in nude rats based on cell cycle inhibition and apoptosis induction. Cancer Lett..

[125] Singh S.K., Banerjee S., Lillard J.W., Singh R. (2016). Expression of CCR5 and its ligand CCL5 in pancreatic cancer. Am. Assoc. Immnol..

[126] Goecke H., Forssmann U., Uguccioni M., Friess H., Conejo-Garcia J.R., Zimmermann A., Baggiolini M., Büchler M.W. (2000). Macrophages infiltrating the tissue in chronic pancreatitis express the chemokine receptor CCR5. Surgery.

[127] Gao Z., Wang X., Wu K., Zhao Y., Hu G. (2010). Pancreatic stellate cells increase the invasion of human pancreatic cancer cells through the stromal cell-derived factor-1/CXCR4 axis. Pancreatology.

[128] Sarmiento L., Frisk G., Anagandula M., Hodik M., Barchetta I., Netanyah E., Cabrera-Rode E., Cilio C.M. (2017). Echovirus 6 infects human exocrine and endocrine pancreatic cells and induces pro-inflammatory innate immune response. Viruses.

[129] Carroll-Anzinger D., Kumar A., Adarichev V., Kashanchi F., Al-Harthi L. (2007). Human immunodeficiency virus-restricted replication in astrocytes and the ability of gamma interferon to modulate this restriction are regulated by a downstream effector of the Wnt signaling pathway. J. Virol..

[130] Brack-Werner R. (1999). Astrocytes: HIV cellular reservoirs and important participants in neuropathogenesis. Aids.

[131] Kanmogne G.D., Kennedy R., Grammas P. (2002). HIV-1 gp120 proteins and gp160 peptides are toxic to brain endothelial cells and neurons: Possible pathway for HIV entry into the brain and HIV-associated dementia. J. Neuropathol. Exp. Neurol..

[132] Gorelick F.S., Pandol S., Jamieson J.D. (2018). Structure-function relationships in the pancreatic acinar cell. Physiology of the Gastrointestinal Tract.

[133] Lodish H., Berk A., Zipursky S., Matsudaira P., Baltimore D., Darnell J. (2000). Folding, Modification, and Degradation of Proteins. Molecular Cell Biology.

[134] Hetz C. (2012). The unfolded protein response: Controlling cell fate decisions under ER stress and beyond. Nat. Rev. Mol. Cell Biol..

[135] Li S., Kong L., Yu X. (2015). The expanding roles of endoplasmic reticulum stress in virus replication and pathogenesis. Crit. Rev. Microbiol..

[136] Nooka S., Ghorpade A. (2018). Organellar stress intersects the astrocyte endoplasmic reticulum, mitochondria and nucleolus in HIV associated neurodegeneration. Cell Death Dis..

[137] Nooka S., Ghorpade A. (2017). HIV-1-associated inflammation and antiretroviral therapy regulate astrocyte endoplasmic reticulum stress responses. Cell Death Discov.

[138] Shah A., Vaidya N.K., Bhat H.K., Kumar A. (2016). HIV-1 gp120 induces type-1 programmed cell death through ER stress employing IRE1α, JNK and AP-1 pathway. Sci. Rep..

[139] Fan Y., He J.J. (2016). HIV-1 Tat induces unfolded protein response and endoplasmic reticulum stress in astrocytes and causes neurotoxicity through glial fibrillary acidic protein (GFAP) activation and aggregation. J. Biol. Chem..

[140] Colli M.L., Paula F.M., Marselli L., Marchetti P., Roivainen M., Eizirik D.L. (2019). Coxsackievirus B tailors the unfolded protein response to favour viral amplification in pancreatic β cells. J. Innate Immun..

[141] Hirota M., Kitagaki M., Itagaki H., Aiba S. (2006). Quantitative measurement of spliced XBP1 mRNA as an indicator of endoplasmic reticulum stress. J. Toxicol. Sci..

[142] Tabas I., Ron D. (2011). Integrating the mechanisms of apoptosis induced by endoplasmic reticulum stress. Nat. Cell Biol..

[143] Saveljeva S., Mc Laughlin S.L., Vandenabeele P., Samali A., Bertrand M.J.M. (2015). Endoplasmic reticulum stress induces ligand-independent TNFR1-mediated necroptosis in L929 cells. Cell Death Dis..

[144] Sano R., Reed J.C. (2013). ER stress-induced cell death mechanisms. Biochim. Biophys. Acta BBA Mol. Cell Res..

[145] Szegezdi E., Logue S.E., Gorman A.M., Samali A. (2006). Mediators of endoplasmic reticulum stress-induced apoptosis. EMBO Rep..

[146] Kara M., Oztas E. (2019). Endoplasmic reticulum stress-mediated cell death. Programmed Cell Death.

[147] Bhat T.A., Chaudhary A.K., Kumar S., O’Malley J., Inigo J.R., Kumar R., Yadav N., Chandra D. (2017). Endoplasmic reticulum-mediated unfolded protein response and mitochondrial apoptosis in cancer. Biochim. Biophys. Acta Rev. Cancer.

[148] Fribley A., Zhang K., Kaufman R.J. (2009). Regulation of apoptosis by the unfolded protein response. Methods Mol. Biol..

[149] Harding H.P., Zhang Y., Bertolotti A., Zeng H., Ron D. (2000). Perk is essential for translational regulation and cell survival during the unfolded protein response. Mol. Cell.

[150] Morishima N., Nakanishi K., Nakano A. (2011). Activating Transcription Factor-6 (ATF6) Mediates Apoptosis with Reduction of Myeloid Cell Leukemia Sequence 1 (Mcl-1) Protein via Induction of WW Domain Binding Protein 1*. J. Biol. Chem..

[151] Bergsbaken T., Fink S.L., Cookson B.T. (2009). Pyroptosis: Host cell death and inflammation. Nat. Rev. Microbiol.

[152] Lebeaupin C., Proics E., De Bieville C., Rousseau D., Bonnafous S., Patouraux S., Adam G., Lavallard V., Rovere C., Le Thuc O. (2015). ER stress induces NLRP3 inflammasome activation and hepatocyte death. Cell Death Dis..

[153] Zhang J., Zhang K., Li Z., Guo B. (2016). ER stress-induced inflammasome activation contributes to hepatic inflammation and steatosis. J. Clin. Cell. Immunol..

[154] Jones B.A., Gores G.J. (1997). Physiology and pathophysiology of apoptosis in epithelial cells of the liver, pancreas, and intestine. Am. J. Physiol. Gastrointest. Liver Physiol..

[155] Herzenberg L.A., De Rosa S.C., Dubs J.G., Roederer M., Anderson M.T., Ela S.W., Deresinski S.C., Herzenberg L.A. (1997). Glutathione deficiency is associated with impaired survival in HIV disease. Proc. Natl. Acad. Sci. USA.

[156] Suthanthiran M., Anderson M.E., Sharma V.K., Meister A. (1990). Glutathione regulates activation-dependent DNA synthesis in highly purified normal human T lymphocytes stimulated via the CD2 and CD3 antigens. Proc. Natl. Acad. Sci. USA.

[157] Sönnerborg A., Carlin G., Åkerlund B., Jarstrand C. (1988). Increased production of malondialdehyde in patients with HIV infection. Scand. J. Infect. Dis..

[158] Reshi M.L., Su Y.-C., Hong J.-R. (2014). RNA viruses: ROS-mediated cell death. Int. J. Cell Biol..

[159] Watanabe L.M., Júnior F.B., Jordão A.A., Navarro A.M. (2016). Influence of HIV infection and the use of antiretroviral therapy on selenium and selenomethionine concentrations and antioxidant protection. Nutrition.

[160] Zhang Y., Wang M., Li H., Zhang H., Shi Y., Wei F., Liu D., Liu K., Chen D. (2012). Accumulation of nuclear and mitochondrial DNA damage in the frontal cortex cells of patients with HIV-associated neurocognitive disorders. Brain Res..

[161] Haughey N.J., Cutler R.G., Tamara A., McArthur J.C., Vargas D.L., Pardo C.A., Turchan J., Nath A., Mattson M.P. (2004). Perturbation of sphingolipid metabolism and ceramide production in HIV-dementia. Ann. Neurol. Off. J. Am. Neurol. Assoc. Child. Neurol. Soc..

[162] Brundu S., Palma L., Picceri G.G., Ligi D., Orlandi C., Galluzzi L., Chiarantini L., Casabianca A., Schiavano G.F., Santi M. (2016). Glutathione depletion is linked with Th2 polarization in mice with a retrovirus-induced immunodeficiency syndrome, murine AIDS: Role of proglutathione molecules as immunotherapeutics. J. Virol..

[163] Kawauchi Y., Suzuki K., Watanabe S., Yamagiwa S., Yoneyama H., Han G.D., Palaniyandi S.S., Veeraveedu P.T., Watanabe K., Kawachi H. (2006). Role of IP-10/CXCL10 in the progression of pancreatitis-like injury in mice after murine retroviral infection. Am. J. Physiol. Gastrointest. Liver Physiol..

[164] Salmen S., Colmenares M., Peterson D.L., Reyes E., Rosales J.D., Berrueta L. (2010). HIV-1 Nef associates with p22-phox, a component of the NADPH oxidase protein complex. Cell. Immunol..

[165] Pandhare J., Dash S., Jones B., Villalta F., Dash C. (2015). A novel role of proline oxidase in HIV-1 envelope glycoprotein-induced neuronal autophagy. J. Biol. Chem..

[166] Gu Y., Wu R.F., Xu Y.C., Flores S.C., Terada L.S. (2001). HIV Tat activates c-Jun amino-terminal kinase through an oxidant-dependent mechanism. Virology.

[167] Banki K., Hutter E., Gonchoroff N.J., Perl A. (1998). Molecular ordering in HIV-induced apoptosis oxidative stress, activation of caspases, and cell survival are regulated by transaldolase. J. Biol. Chem..

[168] Ivanov A.V., Valuev-Elliston V.T., Ivanova O.N., Kochetkov S.N., Starodubova E.S., Bartosch B., Isaguliants M.G. (2016). Oxidative stress during HIV infection: Mechanisms and consequences. Oxidative Med. Cell. Longev..

[169] El-Amine R., Germini D., Zakharova V.V., Tsfasman T., Sheval E.V., Louzada R.A., Dupuy C., Bilhou-Nabera C., Hamade A., Najjar F. (2018). HIV-1 Tat protein induces DNA damage in human peripheral blood B-lymphocytes via mitochondrial ROS production. Redox Biol..

[170] Sakuma Y., Kodama Y., Eguchi T., Uza N., Tsuji Y., Shiokawa M., Maruno T., Kuriyama K., Nishikawa Y., Yamauchi Y. (2018). Chemokine CXCL16 mediates acinar cell necrosis in cerulein induced acute pancreatitis in mice. Sci. Rep..

[171] Gukovskaya A.S., Perkins P., Zaninovic V., Sandoval D., Rutherford R., Fitzsimmons T., Pandol S.J., Poucell-Hatton S. (1996). Mechanisms of cell death after pancreatic duct obstruction in the opossum and the rat. Gastroenterology.

[172] Labarrere C.A., Woods J., Hardin J., Campana G., Ortiz M., Jaeger B., Reichart B., Bonnin J., Currin A., Cosgrove S. (2011). Early prediction of cardiac allograft vasculopathy and heart transplant failure. Am. J. Transplant..

[173] Leurquin-Sterk G., Schepers K., Delhaye M., Goldman S., Verset L., Matos C. (2011). Diffuse pancreatic lesion mimicking autoimmune pancreatitis in an HIV-infected patient: Successful treatment by antiretroviral therapy. Jop. J. Pancreas.

[174] Kaiser A.M., Saluja A.K., Sengupta A., Saluja M., Steer M.L. (1995). Relationship between severity, necrosis, and apoptosis in five models of experimental acute pancreatitis. Am. J. Physiol. Cell Physiol..

[175] Gukovskaya A.S., Gukovsky I. (2011). Which way to die: The regulation of acinar cell death in pancreatitis by mitochondria, calcium, and reactive oxygen species. Gastroenterology.

[176] Bläuer M., Laaninen M., Sand J., Laukkarinen J. (2016). Reciprocal stimulation of pancreatic acinar and stellate cells in a novel long-term in vitro co-culture model. Pancreatology.

[177] Apte M., Haber P., Darby S., Rodgers S., McCaughan G., Korsten M., Pirola R., Wilson J. (1999). Pancreatic stellate cells are activated by proinflammatory cytokines: Implications for pancreatic fibrogenesis. Gut.

[178] Luttenberger T., Schmid-Kotsas A., Menke A., Siech M., Beger H., Adler G., Grünert A., Bachem M.G. (2000). Platelet-derived growth factors stimulate proliferation and extracellular matrix synthesis of pancreatic stellate cells: Implications in pathogenesis of pancreas fibrosis. Lab. Investig..

[179] Schneider E., Schmid-Kotsas A., Zhao J., Weidenbach H., Schmid R.M., Menke A., Adler G., Waltenberger J., Grünert A., Bachem M.G. (2001). Identification of mediators stimulating proliferation and matrix synthesis of rat pancreatic stellate cells. Am. J. Physiol. Cell Physiol..

[180] Shek F.W.-T., Benyon R.C., Walker F.M., McCrudden P.R., Pender S.L.F., Williams E.J., Johnson P.A., Johnson C.D., Bateman A.C., Fine D.R. (2002). Expression of transforming growth factor-β1 by pancreatic stellate cells and its implications for matrix secretion and turnover in chronic pancreatitis. Am. J. Pathol..

[181] Phillips P., Wu M., Kumar R., Doherty E., McCarroll J., Park S., Pirola R.C., Wilson J., Apte M. (2003). Cell migration: A novel aspect of pancreatic stellate cell biology. Gut.

[182] Mews P., Phillips P., Fahmy R., Korsten M., Pirola R., Wilson J., Apte M. (2002). Pancreatic stellate cells respond to inflammatory cytokines: Potential role in chronic pancreatitis. Gut.

[183] Hama K., Ohnishi H., Aoki H., Kita H., Yamamoto H., Osawa H., Sato K., Tamada K., Mashima H., Yasuda H. (2006). Angiotensin II promotes the proliferation of activated pancreatic stellate cells by Smad7 induction through a protein kinase C pathway. Biochem. Biophys. Res. Commun..

[184] National Institute of Alcohol Abuse and Alcoholism Alcohol Facts and Statistics. https://www.niaaa.nih.gov/publications/brochures-and-fact-sheets/alcohol-facts-and-statistics.

[185] Zablotska I.B., Gray R.H., Serwadda D., Nalugoda F., Kigozi G., Sewankambo N., Lutalo T., Mangen F.W., Wawer M. (2006). Alcohol use before sex and HIV acquisition: A longitudinal study in Rakai, Uganda. Aids.

[186] Vagenas P., Ludford K.T., Gonzales P., Peinado J., Cabezas C., Gonzales F., Lama J.R., Sanchez J., Altice F.L., for the Peruvian HIV Sentinel Surveillance Working Group (2014). Being unaware of being HIV-infected is associated with alcohol use disorders and high-risk sexual behaviors among men who have sex with men in Peru. AIDS Behav..

[187] Braithwaite R.S., Bryant K.J. (2010). Influence of alcohol consumption on adherence to and toxicity of antiretroviral therapy and survival. Alcohol Res. Health.

[188] Sansone R.A., Sansone L.A. (2008). Alcohol/Substance misuse and treatment nonadherence: Fatal attraction. Psychiatry (Edgmont).

[189] Santos G.-M., Emenyonu N.I., Bajunirwe F., Mocello A.R., Martin J.N., Vittinghoff E., Bangsberg D.R., Hahn J.A. (2014). Self-reported alcohol abstinence associated with ART initiation among HIV-infected persons in rural Uganda. Drug Alcohol Depend..

[190] Lifson A.R., Demissie W., Tadesse A., Ketema K., May R., Yakob B., Metekia M., Slater L., Shenie T. (2013). Barriers to retention in care as perceived by persons living with HIV in rural Ethiopia: Focus group results and recommended strategies. J. Int. Assoc. Provid. Aids Care.

[191] Kalichman S.C., Amaral C.M., White D., Swetsze C., Kalichman M.O., Cherry C., Eaton L. (2012). Alcohol and adherence to antiretroviral medications: Interactive toxicity beliefs among people living with HIV. J. Assoc. Nurses Aids Care.

[192] Kader R., Seedat S., Govender R., Koch J., Parry C. (2014). Hazardous and harmful use of alcohol and/or other drugs and health status among South African patients attending HIV clinics. AIDS Behav..

[193] Marcellin F., Lions C., Winnock M., Salmon D., Durant J., Spire B., Mora M., Loko M.A., Dabis F., Dominguez S. (2013). Self-reported alcohol abuse in HIV–HCV co-infected patients: A better predictor of HIV virological rebound than physician’s perceptions (HEPAVIH ARNS CO 13 cohort). Addiction.

[194] Kalichman S.C., Grebler T., Amaral C.M., McNerney M., White D., Kalichman M.O., Cherry C., Eaton L. (2014). Viral suppression and antiretroviral medication adherence among alcohol using HIV-positive adults. Int. J. Behav. Med..

[195] Skeer M.R., Mimiaga M.J., Mayer K.H., O’Cleirigh C., Covahey C., Safren S.A. (2012). Patterns of substance use among a large urban cohort of HIV-infected men who have sex with men in primary care. AIDS Behav..

[196] Talamini G., Bassi C., Falconi M., Sartori N., Salvia R., Rigo L., Castagnini A., Di Francesco V., Frulloni L., Bovo P. (1999). Alcohol and smoking as risk factors in chronic pancreatitis and pancreatic cancer. Dig. Dis. Sci..

[197] Lin Y. (2001). Research Committee on Intractable Pancreatic Diseases. Associations of alcohol drinking and nutrient intake with chronic pancreatitis: Findings from a case-control study in Japan. Am. J. Gastroenterol..

[198] Blomgren K.B., Sundström A., Steineck G., Genell S., Sjöstedt S., Wiholm B.-E. (2002). A Swedish case-control network for studies of drug-induced morbidity–acute pancreatitis. Eur. J. Clin. Pharmacol..

[199] Kristiansen L., Grønbæk M., Becker U., Tolstrup J.S. (2008). Risk of pancreatitis according to alcohol drinking habits: A population-based cohort study. Am. J. Epidemiol..

[200] Haber P., Wilson J., Apte M., Korsten M., Pirola R. (1994). Chronic ethanol consumption increases the fragility of rat pancreatic zymogen granules. Gut.

[201] Gorelick F.S. (2003). Alcohol and zymogen activation in the pancreatic acinar cell. Pancreas.

[202] Norton I. (1996). P4502E1 is present in rat pancreas and is induced by chronic ethanol administration. Gastroenterology.

[203] Foster J.R., Idle J.R., Hardwick J.P., Bars R., Scott P., Braganza J.M. (1993). Induction of drug-metabolizing enzymes in human pancreatic cancer and chronic pancreatitis. J. Pathol..

[204] Vonlaufen A., Wilson J.S., Pirola R.C., Apte M.V. (2007). Role of alcohol metabolism in chronic pancreatitis. Alcohol Res. Health.

[205] Yokoyama A., Mizukami T., Matsui T., Yokoyama T., Kimura M., Matsushita S., Higuchi S., Maruyama K. (2013). Genetic Polymorphisms of Alcohol Dehydrogenase-1 B and Aldehyde Dehydrogenase-2 and Liver Cirrhosis, Chronic Calcific Pancreatitis, Diabetes Mellitus, and Hypertension Among J apanese Alcoholic Men. Alcohol. Clin. Exp. Res..

[206] Chiang C.P., Wu C.W., Lee S.P., Chung C.C., Wang C.W., Lee S.L., Nieh S., Yin S.J. (2009). Expression pattern, ethanol-metabolizing activities, and cellular localization of alcohol and aldehyde dehydrogenases in human pancreas: Implications for pathogenesis of alcohol-induced pancreatic injury. Alcohol. Clin. Exp. Res..

[207] Altomare E., Grattagliano I., Vendemiale G., Palmieri V., Palasciano G. (1996). Acute ethanol administration induces oxidative changes in rat pancreatic tissue. Gut.

[208] Hamamoto T., Yamada S., Hirayama C. (1990). Nonoxidative metabolism of ethanol in the pancreas; implication in alcoholic pancreatic damage. Biochem. Pharmacol..

[209] Laposata E.A., Lange L.G. (1986). Presence of nonoxidative ethanol metabolism in human organs commonly damaged by ethanol abuse. Science.

[210] Haber P.S., Apte M.V., Moran C., Applegate T.L., Pirola R.C., Korsten M.A., McCaughan G.W., Wilson J.S. (2004). Non-oxidative metabolism of ethanol by rat pancreatic acini. Pancreatology.

[211] Klochkov A., Kudaravalli P., Sun Y. (2020). Alcoholic pancreatitis. StatPearls [Internet].

[212] Kruger B., Albrecht E., Lerch M.M. (2000). The role of intracellular calcium signaling in premature protease activation and the onset of pancreatitis. Am. J. Pathol.

[213] Gorelick F.S., Thrower E. (2009). The acinar cell and early pancreatitis responses. Clin. Gastroenterol. Hepatol..

[214] Clemens D.L., Schneider K.J., Arkfeld C.K., Grode J.R., Wells M.A., Singh S. (2016). Alcoholic pancreatitis: New insights into the pathogenesis and treatment. World J. Gastrointest. Pathophysiol..

[215] Gerasimenko J.V., Gryshchenko O., Ferdek P.E., Stapleton E., Hébert T.O., Bychkova S., Peng S., Begg M., Gerasimenko O.V., Petersen O.H. (2013). Ca2+ release-activated Ca2+ channel blockade as a potential tool in antipancreatitis therapy. Proc. Natl. Acad. Sci. USA.

[216] Criddle D.N., Murphy J., Fistetto G., Barrow S., Tepikin A.V., Neoptolemos J.P., Sutton R., Petersen O.H. (2006). Fatty acid ethyl esters cause pancreatic calcium toxicity via inositol trisphosphate receptors and loss of ATP synthesis. Gastroenterology.

[217] Apte M.V., Wilson J.S., Korsten M.A. (1997). Alcohol-related pancreatic damage: Mechanisms and treatment. Alcohol Health Res. World.

[218] Haber P.S., Wilson J.S., Apte M.V., Pirola R.C. (1993). Fatty acid ethyl esters increase rat pancreatic lysosomal fragility. J. Lab. Clin. Med..

[219] Talukdar R., Sareen A., Zhu H., Yuan Z., Dixit A., Cheema H., George J., Barlass U., Sah R., Garg S.K. (2016). Release of cathepsin B in cytosol causes cell death in acute pancreatitis. Gastroenterology.

[220] Sankaran H., Lewin M.B., Wong A., Deveney C.W., Wendland M.F., Leimgruber R.M., Geokas M.C. (1985). Irreversible inhibition by acetaldehyde of cholecystokinin-induced amylase secretion from isolated rat pancreatic acini. Biochem. Pharmacol..

[221] Casini A., Galli A., Pignalosa P., Frulloni L., Grappone C., Milani S., Pederzoli P., Cavallini G., Surrenti C. (2000). Collagen type I synthesized by pancreatic periacinar stellate cells (PSC) co-localizes with lipid peroxidation-derived aldehydes in chronic alcoholic pancreatitis. J. Pathol..

[222] Chadwick S.R., Lajoie P. (2019). Endoplasmic reticulum stress coping mechanisms and lifespan regulation in health and diseases. Front. Cell Dev. Biol..

[223] Ji C., Kaplowitz N. (2003). Betaine decreases hyperhomocysteinemia, endoplasmic reticulum stress, and liver injury in alcohol-fed mice. Gastroenterology.

[224] Lugea A., Tischler D., Nguyen J., Gong J., Gukovsky I., French S.W., Gorelick F.S., Pandol S.J. (2011). Adaptive unfolded protein response attenuates alcohol-induced pancreatic damage. Gastroenterology.

[225] Pandol S.J., Gorelick F.S., Gerloff A., Lugea A. (2010). Alcohol abuse, endoplasmic reticulum stress and pancreatitis. Dig. Dis..

[226] Lugea A., Tischler D., Nguyen J., Gong J., Gukovsky I., French S.W., Gorelick F.S., Pandol S.J. (2011). Basic-liver, pancreas, and biliary tract. Gastroenterology.

[227] Mareninova O.A., Hermann K., French S.W., O’Konski M.S., Pandol S.J., Webster P., Erickson A.H., Katunuma N., Gorelick F.S., Gukovsky I. (2009). Impaired autophagic flux mediates acinar cell vacuole formation and trypsinogen activation in rodent models of acute pancreatitis. J. Clin. Investig..

[228] Wang Y.-L., Hu R., Lugea A., Gukovsky I., Smoot D., Gukovskaya A.S., Pandol S.J. (2006). Ethanol feeding alters death signaling in the pancreas. Pancreas.

[229] Apte M.V., Pirola R., Wilson J.S. (2005). Molecular mechanisms of alcoholic pancreatitis. Dig. Dis..

[230] Apte M., Wilson J., Korsten M., McCaughan G., Haber P., Pirola R. (1995). Effects of ethanol and protein deficiency on pancreatic digestive and lysosomal enzymes. Gut.

[231] Ponnappa B.C., Hoek J.B., Waring A.J., Rubin E. (1987). Effect of ethanol on amylase secretion and cellular calcium homeostasis in pancreatic acini from normal and ethanol-fed rats. Biochem. Pharmacol..

[232] Siegmund E., Lüthen F., Kunert J., Weber H. (2004). Ethanol modifies the actin cytoskeleton in rat pancreatic acinar cells–comparison with effects of CCK. Pancreatology.

[233] Wilson J.S., Korsten M.A., Apte M.V., Thomas M.C., Haber P.S., Pirola R.C. (1990). Both ethanol consumption and protein deficiency increase the fragility of pancreatic lysosomes. J. Lab. Clin. Med..

[234] Duko B., Ayalew M., Ayano G. (2019). The prevalence of alcohol use disorders among people living with HIV/AIDS: A systematic review and meta-analysis. Subst. Abus. Treat. Prev. Policy.

[235] Apte M.V., Pirola R.C., Wilson J.S. (2016). Alcohol and the pancreas. Pancreapedia Exocrine Pancreas Knowl. Base.

[236] Wang X., Douglas S.D., Metzger D.S., Guo C.J., Li Y., O’Brien C.P., Song L., Davis-Vogal A., Ho W.Z. (2002). Alcohol potentiates HIV-1 infection of human blood mononuclear phagocytes. Alcohol. Clin. Exp. Res..

[237] Ambade A., Lowe P., Kodys K., Catalano D., Gyongyosi B., Cho Y., Iracheta-Vellve A., Adejumo A., Saha B., Calenda C. (2019). Pharmacological inhibition of CCR2/5 signaling prevents and reverses alcohol-induced liver damage, steatosis, and inflammation in mice. Hepatology.

[238] Liu X., Zha J., Nishitani J., Chen H., Zack J.A. (2003). HIV-1 infection in peripheral blood lymphocytes (PBLs) exposed to alcohol. Virology.

[239] Wilen C., Tilton J., Doms R. (2012). HIV: Cell Binding and Entry.

[240] Miyauchi K., Kim Y., Latinovic O., Morozov V., Melikyan G.B. (2009). HIV enters cells via endocytosis and dynamin-dependent fusion with endosomes. Cell.

[241] Maréchal V., Clavel F., Heard J.M., Schwartz O. (1998). Cytosolic Gag p24 as an index of productive entry of human immunodeficiency virus type 1. J. Virol..

[242] Cossart P., Helenius A. (2014). Endocytosis of viruses and bacteria. Cold Spring Harb. Perspect. Biol..

[243] Fredericksen B.L., Wei B.L., Yao J., Luo T., Garcia J.V. (2002). Inhibition of Endosomal/Lysosomal Degradation Increases the Infectivity of Human Immunodeficiency Virus. J. Virol..

[244] Kharbanda K.K., McVicker D.L., Zetterman R.K., MacDonald R.G., Donohue T.M. (1997). Flow cytometric analysis of vesicular pH in rat hepatocytes after ethanol administration. Hepatology.

[245] Donohue T.M., Osna N.A. (2003). Intracellular proteolytic systems in alcohol-induced tissue injury. Alcohol Res. Health.

[246] Donohue T.M., Osna N.A., Kharbanda K.K., Thomes P.G. (2019). Lysosome and proteasome dysfunction in alcohol-induced liver injury. Liver Res..

[247] Li Y., Chen M., Xu Y., Yu X., Xiong T., Du M., Sun J., Liu L., Tang Y., Yao P. (2016). Iron-mediated lysosomal membrane permeabilization in ethanol-induced hepatic oxidative damage and apoptosis: Protective effects of quercetin. Oxidative Med. Cell. Longev..

[248] Jajte J., Stetkiewicz J., Wronska-Nofer T. (2003). Combined exposure to m-xylene and ethanol: Oxidative stress in the rat liver. Int. J. Occup. Med. Environ. Health.

[249] Curry-McCoy T.V., Osna N.A., Nanji A.A., Donohue T.M. (2010). Chronic ethanol consumption results in atypical liver injury in copper/zinc superoxide dismutase deficient mice. Alcohol. Clin. Exp. Res..

[250] Doitsh G., Galloway N.L., Geng X., Yang Z., Monroe K.M., Zepeda O., Hunt P.W., Hatano H., Sowinski S., Muñoz-Arias I. (2014). Cell death by pyroptosis drives CD4 T-cell depletion in HIV-1 infection. Nature.

[251] Plymale D.R., Tang D.S., Comardelle A.M., Fermin C.D., Lewis D.E., Garry R.F. (1999). Both necrosis and apoptosis contribute to HIV-1-induced killing of CD4 cells. Aids.

[252] Bhatia M. (2004). Apoptosis versus necrosis in acute pancreatitis. Am. J. Physiol. Gastrointest. Liver Physiol..

[253] Kim Y., Anderson J.L., Lewin S.R. (2018). Getting the “kill” into “shock and kill”: Strategies to eliminate latent HIV. Cell Host Microbe.

[254] Tran T.-A., De Herve M.-g.D.G., Hendel-Chavez H., Dembele B., Le Névot E., Abbed K., Pallier C., Goujard C., Gasnault J., Delfraissy J.-F. (2008). Resting regulatory CD4 T cells: A site of HIV persistence in patients on long-term effective antiretroviral therapy. PLoS ONE.

[255] Rukobia Fostemsavir. https://www.rukobiahcp.com/mechanism-of-action/.

[256] Woollard S.M., Kanmogne G.D. (2015). Maraviroc: A review of its use in HIV infection and beyond. Drug Des. Dev. Ther..

[257] CytoDyn CytoDyn Seeks UK Approval of Leronlimab for HIV and COVID-19. https://www.cytodyn.com/newsroom/press-releases/detail/457/cytodyn-seeks-uk-approval-of-leronlimab-for-hiv-and-covid-19.

[258] Pascua-Maestro R., Diez-Hermano S., Lillo C., Ganfornina M.D., Sanchez D. (2017). Protecting cells by protecting their vulnerable lysosomes: Identification of a new mechanism for preserving lysosomal functional integrity upon oxidative stress. PLoS Genet..

[259] Sun X.-Y., Wang J.-M., Ouyang J.-M., Kuang L. (2018). Antioxidant activities and repair effects on oxidatively damaged HK-2 cells of tea polysaccharides with different molecular weights. Oxidative Med. Cell. Longev..

[260] Song S.B., Hwang E.S. (2020). High Levels of ROS Impair Lysosomal Acidity and Autophagy Flux in Glucose-Deprived Fibroblasts by Activating ATM and Erk Pathways. Biomolecules.

[261] Zhang W., Li X., Wang S., Chen Y., Liu H. (2020). Regulation of TFEB activity and its potential as a therapeutic target against kidney diseases. Cell Death Discov..

[262] Wang F., Gómez-Sintes R., Boya P. (2018). Lysosomal membrane permeabilization and cell death. Traffic.

[263] New-Aaron M., Ganesan M., Dagur R.S., Kharbanda K.K., Poluektova L.Y., Osna N.A. (2020). Obeticholic acid attenuates human immunodeficiency virus/alcohol metabolism-induced pro-fibrotic activation in liver cells. World J. Hepatol..

[264] Zhou X., Xie L., Bergmann F., Endris V., Strobel O., Büchler M.W., Kroemer G., Hackert T., Fortunato F. (2017). The bile acid receptor FXR attenuates acinar cell autophagy in chronic pancreatitis. Cell Death Discov..

[265] Lee J., Lee K., Lee J., Lee K., Jang K., Heo J., Choi S., Kim Y., Rhee J. (2011). Farnesoid X receptor, overexpressed in pancreatic cancer with lymph node metastasis promotes cell migration and invasion. Br. J. Cancer.

[266] Shehzad A., Rehman G., Lee Y.S. (2013). Curcumin in inflammatory diseases. Biofactors.

[267] Aggarwal B.B. (2010). Targeting inflammation-induced obesity and metabolic diseases by curcumin and other nutraceuticals. Annu. Rev. Nutr..

[268] Asaumi H., Watanabe S., Taguchi M., Tashiro M., Nagashio Y., Nomiyama Y., Nakamura H., Otsuki M. (2006). Green tea polyphenol (–)-epigallocatechin-3-gallate inhibits ethanol-induced activation of pancreatic stellate cells. Eur. J. Clin. Investig..

